# Exogenous SA- and IAA-Mediated Regulation of Silique Growth and Development in *Isatis indigotica*

**DOI:** 10.3390/life16101587

**Published:** 2026-09-23

**Authors:** Dong Liu, Jinjin Meng, Menghu Wang, Qianqian Jiang, Pan Wang, Xijia Jiu, Tao Du, Xiangsong Meng

**Affiliations:** 1 School of Chinese Materia Medica and Health, Bozhou University, Bozhou 236800, China; liudong804214@163.com (D.L.);; 2 School of Pharmacy, Gansu Health Vocational College, Lanzhou New Area, Lanzhou 730207, China; 3 Northwest Institute of Plateau Biology, Chinese Academy of Sciences, Xining 810008, China; 4 School of Pharmacy, Gansu University of Chinese Medicine, Chengguan District, Lanzhou 730000, China

**Keywords:** *Isatis indigotica* Fort., silique development, salicylic acid, indole-3-acetic acid, carbohydrate metabolism

## Abstract

*Isatis indigotica* Fort.—a well-known medicinal cruciferous species—is the source plant of *Isatidis Radix* and *Folium Isatidis*, and is widely cultivated for medicinal raw material. The silique serves as the propagative organ for seed production of *I. indigotica*, and its developmental performance directly determines the plant’s seed quality and seed multiplication efficiency. To decipher the physiological and molecular mechanisms by which exogenous salicylic acid (SA) and indole-3-acetic acid (IAA) modulate silique development, we performed a field experiment using the cultivar ‘Songming No. 1’. Plants were foliar-sprayed with 2 mmol·L^−1^ SA at the full-bloom stage and 0.571 mmol·L^−1^ IAA at the flower-bud stage, with distilled water treatment serving as the control (CK). Silique samples were collected at five key developmental stages: formation, growth, expansion, browning maturation, and full maturation. We measured nutrient contents, carbohydrate-metabolizing enzyme activities, and endogenous phytohormone levels, and performed integrated transcriptomic and untargeted metabolomic analyses. Exogenous SA and IAA substantially reshaped the accumulation patterns of sucrose, starch, soluble proteins, and other nutrients. The activities of invertase, pyruvate kinase (PK), glucose-6-phosphate dehydrogenase (G6PDH), and soluble starch synthase (SSS) were significantly altered by hormone treatments; SA accelerated metabolic processes in early-stage siliques. Transcriptomic screening identified nine SA-responsive genes and one IAA-responsive candidate gene. SA mainly perturbed carbohydrate and amino acid metabolic pathways, whereas IAA modulated terpenoid and polyketide biosynthetic pathways. A total of 7028 metabolites were annotated, showing that SA exerted stronger effects on carbohydrate metabolism than IAA. Nine-quadrant correlation analysis identified multiple gene–metabolite modules associated with silique development. Collectively, this study characterizes the physiological and multi-omics responses of *I. indigotica* siliques to exogenous SA and IAA. The physiological and molecular alterations observed here are consistent with the phenotypic outcomes reported under identical experimental treatments. This work provides valuable resources for exploring hormone-mediated high-quality seed propagation and candidate gene mining in *I. indigotica*.

## 1. Introduction

*Isatis indigotica* Fort. is a biennial medicinal herb of the genus *Isatis* in the Brassicaceae family. Its roots and leaves are officially recorded in the Chinese Pharmacopoeia (2025 edition) as the traditional Chinese medicines *Radix Isatidis* and *Folium Isatidis*, respectively, and it is widely cultivated for medicinal material production.

Siliques serve as the primary sowing propagules in practical *I. indigotica* production. Field investigations have identified prominent agronomic bottlenecks in large-scale cultivation, including unstable seedling emergence rates, heterogeneous seedling growth, and a considerable proportion of empty siliques during maturation. Silique developmental status directly determines internal seed vigor and subsequent field seedling performance. Seed quality defects derived from abnormal silique development have become a major factor restricting the standardized and mechanized cultivation of *I. indigotica*. Therefore, exploring the regulatory mechanisms underlying seed propagation is of great practical significance for stabilizing seedling quality and ensuring the sustainable production of *I. indigotica*-derived medicinal materials.

Salicylic acid (SA), an endogenous phenolic signaling compound, participates extensively in plant growth and reproductive development. SA regulates organ morphogenesis by modulating cell division and cell expansion [1], modulates seed enzymatic activities and storage reserve catabolism to alleviate seed dormancy and accelerate germination [2], and exhibits concentration-dependent dual effects on reproductive organ development [3,4]. Previous studies have largely focused on the roles of SA in disease resistance [5] and abiotic stress tolerance [6]; however, the molecular mechanisms through which SA modulates reproductive organ development remain poorly understood.

Indole-3-acetic acid (IAA), a core indole-derived auxin, plays central regulatory roles in fruit development, organ morphogenesis [7], and seed developmental processes [8,9]. IAA mediates cell elongation [10], promotes early fruit expansion [11], and participates in organ senescence and abscission [12] as well as apical dominance [13,14]. Notably, its regulatory effects on reproductive organs are highly dependent on the hormone concentration and application stage, with inappropriate timing or excessive concentrations commonly disrupting fruit and seed development. To date, mechanistic research into IAA-modulated silique development in Brassicaceae species remains limited.

Transcriptomics and metabolomics are powerful tools for dissecting molecular regulatory networks underlying plant reproductive organ development, enabling systematic elucidation of correlations among hormone-modulated gene expression, substance accumulation, and metabolite reprogramming [15,16], and supporting the screening of candidate genes [17] and metabolic biomarkers associated with fruit and seed development [18].

In our preliminary experiments, biennial *I. indigotica* plants were foliarly sprayed with six plant growth regulators—namely, diethyl aminoethyl hexanoate (DA-6), brassinosteroid (BR), gibberellin (GA), methyl jasmonate (MeJA), IAA, and SA—at five critical growth stages: the flower-bud stage, full-bloom stage, 4 days post-anthesis (DPA), 10 DPA, and 16 DPA. Core agronomic traits, including fruit morphology, yield component characteristics, and seed germination rate, were systematically determined. Previous results have revealed that foliar application of 2 mmol·L^−1^ SA at the full-bloom stage significantly improved the seed yield and quality of *I. indigotica*, whereas 0.571 mmol·L^−1^ IAA applied at the flower-bud stage inhibited silique development and deteriorated seed performance [19]. However, these preliminary studies only focused on phenotypic and yield assessments, leaving the physiological and molecular mechanisms of exogenous SA and IAA regulating silique development in *I. indigotica* unclarified.

Accordingly, this study performed foliar spraying of IAA at the flower-bud stage and SA at the full-bloom stage on *I. indigotica* plants. Silique samples were collected at key developmental stages to quantify nutrient contents, the activities of core carbohydrate-metabolizing enzymes, and endogenous phytohormone levels. Furthermore, integrated transcriptomic and untargeted metabolomic analyses were performed to explore the regulatory networks of silique development. This study aims to clarify the physiological basis and molecular mechanisms of SA- and IAA-modulated silique development, and to screen key candidate genes and differential metabolites closely associated with silique growth and development. The findings provide a theoretical foundation and empirical data for elucidating hormone regulatory mechanisms and optimizing phytohormone application for high-quality seed propagation of *I. indigotica*.

## 2. Materials and Methods

### 2.1. Plant Materials

The plant material used in this study was the *I. indigotica* Fort. cultivar ‘Songming No. 1’. Field experiments were carried out at the Hezheng Medicinal Botanical Garden, Gansu University of Chinese Medicine (35°15′48″ N, 103°24′21″ E). The garden is located at an altitude of 2430 m, with a mean annual precipitation of 660 mm, a frost-free period of 110 d, and an average annual temperature of 5.1 °C. The annual maximum and minimum air temperatures were 29.0 °C and −20.4 °C, respectively. The experimental field had dark loessial soil, and the plot was fallow prior to planting.

On 23 May 2023, silique-borne seeds of *I. indigotica* Fort. cv. ‘Songming No. 1’ were sown in rows with a row spacing of 50 cm and a sowing depth of 3–5 cm. Thinning was performed when seedlings produced six true leaves to achieve a plant spacing of 10 cm. Conventional field management following local agronomic practices was applied throughout the growing season. After plant regreening on 15 April 2024, surplus plants were removed to adjust the plant spacing to 50 cm. Compound fertilizer (N-P_2_O_5_-K_2_O, 15-15-15) was applied at 300 kg·hm^−2^ during the regreening and bolting stage. No additional artificial agronomic measures were applied during the experimental period.

### 2.2. Experimental Design

Based on our group’s prior trials involving six phytohormones—namely, diethyl aminoethyl hexanoate (DA-6), brassinosteroid (BR), gibberellin (GA), methyl jasmonate (MeJA), IAA, and SA, each evaluated across three concentration gradients—comprehensive screening was performed using agronomic indices including per-plant yield, 1000-seed weight, seed number per silique, silique traits, and seed germination rate. These assays revealed that foliar application of 2 mmol·L^−1^ SA at the full-bloom stage markedly improves seed yield and seed quality of *I. indigotica*, whereas 100 mg·L^−1^ IAA sprayed at the flower-bud stage impairs silique development and deteriorates seed quality. Accordingly, the foliar spray treatments were established as follows: 0.571 mmol·L^−1^ indole-3-acetic acid (IAA) was applied at the flower-bud stage (27 April), and 2 mmol·L^−1^ salicylic acid (SA) was sprayed at the full-bloom stage (16 May). Plants receiving equivalent volumes of distilled water served as the control group (CK). Each experimental plot covered an area of 11 m^2^ with 40 individual plants, and three biological replicates were arranged for all treatments. Foliar spraying was terminated once fine droplets uniformly coated the surfaces of leaves and flower buds. Uniform agronomic practices were implemented across all experimental units throughout the field trial.

According to the developmental staging criteria for *I. indigotica* siliques, samples were collected at five key developmental phases: silique formation, silique growth, silique expansion, silique browning maturation, and silique full maturation (Table 1). For each plot, five healthy plants with consistent growth were randomly selected. Intact disease- and pest-free siliques were harvested from the proximal half-segment of primary lateral branches on the outer canopy. Samples from the same plot were pooled to constitute one biological replicate. Fresh samples were immediately snap-frozen in liquid nitrogen and stored at −80 °C in an ultra-low-temperature freezer for subsequent physiological measurement, transcriptome sequencing, and metabolomic profiling. All physiological, transcriptomic, and metabolomic analyses were performed using the above-mentioned three biological replicates.

Phenotypic evaluations, including silique size, seed-set rate, seed yield, and seed germination performance under identical hormone treatments, have been reported in our previously published study (https://doi.org/10.13422/j.cnki.syfjx.20260416). The present study focuses on physiological, transcriptomic, and untargeted metabolomic profiling of silique samples collected from the same experimental plots, without repeating those published phenotypic measurements.

### 2.3. Experimental Procedures

#### 2.3.1. Determination of Nutrient Content in *Isatis indigotica* Siliques

Frozen silique samples from IAA-treated, SA-treated, and CK groups across five developmental stages were retrieved from the −80 °C freezer. The contents of soluble protein (SP), lignin, and cellulose (CLL) were determined using commercial assay kits (Jiangxi Hailian Biotechnology Co., Ltd., Nanchang, Jiangxi, China). Sucrose, starch, soluble sugar (SS), and resistant starch (RS) were assayed with kits from Solarbio Science & Technology Co., Ltd. (Beijing, China). The core assay principles were as follows: soluble protein was quantified via the Coomassie Brilliant Blue G-250 staining method; lignin and cellulose were measured using concentrated sulfuric acid hydrolysis colorimetry; carbohydrate-related indices were detected using the anthrone–sulfuric acid colorimetric method. All operations strictly followed the manufacturer’s instructions. Three technical replicates were set for each biological replicate.

#### 2.3.2. Assays of Carbohydrate-Metabolizing Enzymes and Endogenous Phytohormones in *Isatis indigotica* Siliques

Frozen silique samples of IAA-treated, SA-treated, and CK groups covering five developmental stages were taken from the −80 °C ultra-low-temperature freezer. Enzymatic activities of sucrase, sucrose synthase (cleavage direction, SS-I), sucrose synthase (synthetic direction, SS-II), soluble starch synthase (SSS), and starch-branching enzyme (SBE) were measured using commercial kits (Jiangxi Hailian Biotechnology Co., Ltd.). Pyruvate kinase (PK) and glucose-6-phosphate dehydrogenase (G6PDH) activities were determined with kits purchased from Solarbio Science & Technology Co., Ltd. (Beijing, China). Endogenous phytohormones, including indole-3-acetic acid (IAA), gibberellin (GA), abscisic acid (ABA), and salicylic acid, were quantified via enzyme-linked immunosorbent assay (ELISA) using kits from Shanghai Qincheng Biotechnology (Shanghai, China). Enzyme activities were determined spectrophotometrically. Endogenous hormone quantification adopted a solid-phase antibody-based competitive ELISA, in which endogenous hormones in samples compete with enzyme-conjugated hormones for antibody-binding sites coated on microplates. Hormone concentrations were calculated according to color-development absorbance values. All experimental procedures were performed strictly following the manufacturer’s protocols. Three technical replicates were conducted for each biological replicate.

#### 2.3.3. Transcriptome Sequencing and qRT-PCR Validation of *Isatis indigotica* Siliques

##### RNA Isolation, Library Construction, and Sequencing

Total RNA was extracted from silique samples of CK, SA-treated, and IAA-treated groups across five developmental stages. RNA concentration and purity were evaluated with a NanoDrop 2000 spectrophotometer (Thermo Fisher Scientific, Wilmington, DE, USA). RNA degradation and integrity were assessed via 1% agarose gel electrophoresis, and the RNA quality number (RQN) was determined using an Agilent 5300 system (Agilent Technologies, Santa Clara, CA, USA). Messenger RNA was enriched with oligo-dT magnetic beads and fragmented using fragmentation buffer. First-strand cDNA was synthesized via reverse transcription, followed by second-strand cDNA synthesis. Adapter sequences were ligated to double-stranded cDNA fragments. Ligation products were purified and subjected to fragment size selection. Size-selected fragments were amplified via PCR and purified to construct sequencing libraries, which were subsequently subjected to high-throughput sequencing.

##### Sequencing Data Quality Control and Bioinformatic Analyses

Raw sequencing reads were preprocessed to remove adapters and low-quality reads, generating clean reads for downstream analysis. Since no public reference genome is available for *I. indigotica*, clean reads were de novo assembled using the Trinity (v2.11.0) software to obtain unigenes as reference transcripts. Clean reads were then mapped to the assembled unigene set. Gene expression abundance was calculated as FPKM values. Differential expression analysis was performed using DESeq2 (v1.38.0); genes satisfying thresholds of |log_2_FC| ≥ 2 and *p* < 0.05 were defined as differentially expressed genes (DEGs). Unigenes were functionally annotated against multiple databases, including GO, KEGG, EggNOG, NR, Swiss-Prot, and Pfam.

Transcriptome de novo assembly quality metrics: The assembled unigene statistics were as follows: total unigene number, N50, N90, mean length, maximum length, minimum length, GC content, and mapping rate of clean reads.All assembled transcript sequences were longer than 200 bp. Q20 and Q30 values of each sequencing library were calculated to evaluate sequencing base-calling quality.

##### qRT-PCR Validation

Five differentially expressed transcripts were selected for qRT-PCR validation, with Actin used as the internal reference gene. Primer sequences are listed in Table 2. All primer pairs were verified via gradient PCR, with amplification efficiencies ranging from 90% to 110%. First-strand cDNA was synthesized using the EntiLink^TM^ 1st Strand cDNA Synthesis Kit (ELK Biotechnology Co., Ltd., Wuhan, China), and qRT-PCR was carried out with the Fast qPCR Mix Kit (ELK Biotechnology Co., Ltd., Wuhan, China).

The Dnase treatment reaction for RNA was performed in a 15 μL system and incubated at 72 °C for 5 min. The cDNA synthesis system (total volume 21 μL) was incubated at 37 °C for 60 min, and the reaction was terminated at 85 °C for 5 min. The qRT-PCR amplification system (15 μL) contained 10 μL 2× Master Mix, 1 μL 2.5 μmol·L^−1^ primer working solution, 1 μL cDNA template, 2 μL ddH_2_O, and 1 μL Rox reference dye. The thermal profile was set as follows: initial denaturation at 95 °C for 3 min; 40 cycles of 95 °C for 10 s, 58 °C for 30 s, and 72 °C for 30 s. Relative gene expression was calculated using the 2^−ΔΔCT^ method.

#### 2.3.4. Untargeted Metabolomic Profiling of *Isatis indigotica* Siliques

##### Metabolite Extraction and Detection

Silique samples stored at −80 °C were vacuum freeze-dried for 63 h. Dried tissues were ground thoroughly into a fine powder using a tissue grinder (30 Hz, 1.5 min; Retsch GmbH, Haan, Germany). Briefly, 50 mg of pulverized sample was accurately weighed, and 1200 μL of pre-chilled extraction solvent (methanol–acetonitrile–ultrapure water; volume ratio 2:2:1) was added for metabolite extraction after vigorous vortexing. The mixture was centrifuged at 12,000 rpm at 4 °C for 3 min. The supernatant was collected and filtered through a 0.22 μm organic-phase microporous membrane prior to instrumental analysis. Quality control (QC) samples were prepared by pooling equal aliquots from all individual samples and interspersed across the analytical sequence to evaluate instrument stability.

##### Metabolomic Data Analysis

Principal component analysis (PCA) and orthogonal partial least-squares discriminant analysis (OPLS-DA) were performed on the dataset. Differentially accumulated metabolites (DAMs) were screened with thresholds of |log_2_FC| ≥ 1 and VIP > 1. Metabolite annotation was performed by matching accurate mass-to-charge ratios (*m*/*z*), fragment spectra, and retention times against public metabolomic databases, including METLIN, HMDB, and LipidMaps. QC samples were used to monitor instrument stability, and features with RSD > 30% across QC samples were removed before downstream statistical analysis.

### 2.4. Integrated Transcriptome–Metabolome Analysis

DEGs were screened using the thresholds |log_2_FC| ≥ 2 and *p* < 0.05. Candidate genes responsive to exogenous IAA or SA during *I. indigotica* silique development were identified via computational inference. DAMs were filtered with the thresholds |log_2_FC| ≥ 1 and VIP > 1 to obtain metabolites affected by IAA or SA treatments. Correlation analysis between IAA-/SA-responsive DEGs and DAMs was implemented in R (v4.2.1). A nine-quadrant plot was generated to identify key gene–metabolite association modules.

### 2.5. Statistical Data Analysis

Microsoft Excel 2019 was used for raw data sorting. Data processing and graphical visualization were performed in OriginPro 2021. One-way analysis of variance (ANOVA) was used to assess inter-group differences, followed by Duncan’s multiple range test for post hoc comparisons. Differences at *p* < 0.05 were considered statistically significant, whereas *p* < 0.01 indicated highly significant differences.

## 3. Results

### 3.1. Effects of Exogenous IAA and SA on Nutrient Content in Isatis indigotica Siliques

The contents of seven nutritional substances—namely, sucrose (Figure 1A), soluble sugar (Figure 1B), starch (Figure 1C), amylopectin (Figure 1D), lignin (Figure 1E), cellulose (Figure 1F), and soluble protein (Figure 1G)—in *I. indigotica* siliques under control (CK), SA, and IAA treatments across five developmental stages were determined (Figure 1). Sucrose content exhibited a continuous upward trend throughout silique development. At the T1, T2, T4, and T5 stages, the sucrose content in the CK group was significantly higher than that in the SA- and IAA-treated groups (*p* < 0.01). Only at the T3 silique expansion stage did SA treatment result in higher sucrose accumulation compared with CK and IAA treatments.

The soluble sugar content in the CK group increased first and then decreased, peaking at 56.47 mg·g^−1^ at the T2 silique growth stage. In contrast, soluble sugar levels in SA- and IAA-treated groups declined continuously during silique development. Starch accumulation varied substantially among treatments at different developmental stages. At T2 and T3, the starch content under SA treatment was 1–2-fold higher than that in the IAA and CK groups. From T4 to T5, starch contents in all treatments decreased significantly relative to T3 (*p* < 0.05). Amylopectin content gradually decreased from T1 to T5, with no significant differences observed among treatment groups.

For all experimental groups, lignin content increased from T1 to T3, decreased at T4, and increased again at T5. Cellulose accumulation exhibited distinct treatment-specific patterns. In the SA- and IAA-treated groups, cellulose content decreased continuously from T1 to T4 and increased by 50 mg·g^−1^ and 30 mg·g^−1^ at T5 compared with T4, respectively. In the CK group, cellulose content increased steadily from T1 to T4 and declined at T5. Notably, cellulose content in the CK group from T3 to T5 was 3–6 times higher than that in the SA and IAA groups.

Soluble protein content in all treatments increased from T1 to T2, peaked at T2, and then decreased consistently from T2 to T5. The reduction rate of soluble protein under SA treatment was significantly greater than that under CK and IAA treatments.

### 3.2. Effects of Exogenous IAA and SA on Physiologically Active Substances and Endogenous Phytohormones in Isatis indigotica Siliques

Eleven physiological indices, including key enzymes involved in sucrose metabolism, starch biosynthesis, and energy metabolism, as well as the endogenous phytohormones GA, IAA, ABA, and SA, were quantified across five silique developmental stages (Figure 2). Sucrase activity (Figure 2A) generally decreased from T1 to T4 and increased from T4 to T5. During T1–T4, sucrase activities in SA- and IAA-treated groups were markedly higher than those in the CK group, whereas the CK group exhibited a two-fold higher sucrase activity than the SA and IAA groups at T5.

Sucrose synthase (cleavage direction, SS-I) activity (Figure 2B) under SA treatment differed significantly from that under the CK and IAA treatments at all developmental stages. The SA group showed higher SS-I activity than the other two groups at T1, T3, and T4; at T3, SS-I activity in the SA group was 2.7-fold that in the IAA group and 1.3-fold that in the CK group. Sucrose synthase (synthetic direction, SS-II) activity (Figure 2C) under SA treatment increased continuously with silique development, while SS-II activities in the CK and IAA groups increased initially and then declined. Highly significant differences in SS-II activity were detected between the SA group and the other two groups at T1, T2, T4, and T5 (*p* < 0.01).

Pyruvate kinase (PK) activity (Figure 2D) in all groups followed an increasing then decreasing trend. PK activities in the SA and IAA groups were higher than those in CK from T1 to T4. At T5, PK activity was ranked as follows: IAA (2045.73 U·g^−1^) > CK (866.53 U·g^−1^) > SA (544.93 U·g^−1^). Glucose-6-phosphate dehydrogenase (G6PDH) activity (Figure 2E) in the SA- and IAA-treated groups was significantly higher than that in the CK group throughout T1–T4; particularly at T2–T3, G6PDH activity under SA treatment reached three times the level of the CK group.

The overall soluble starch synthase (SSS) activity (Figure 2F) ranking among treatments was SA > CK > IAA. SSS activity remained above 1980 nmol·min^−1^·g^−1^ from T1 to T4 and dropped sharply to below 550 nmol·min^−1^·g^−1^ at T5, with no significant inter-group differences at the T5 stage. Starch-branching enzyme (SBE) activity (Figure 2G) decreased progressively during silique development.

For endogenous phytohormones, SA and IAA treatments induced higher endogenous GA (Figure 3A) and ABA contents (Figure 3B) than CK during T1–T3, while the CK group possessed higher GA and ABA levels than the hormone-treated groups at T4–T5. Endogenous SA content (Figure 3C) in the SA- and IAA-treated groups was significantly higher than that in CK at T2 and T3, whereas no obvious inter-group differences in endogenous SA were observed at T1, T4, and T5.

### 3.3. Transcriptome Sequencing Analysis

#### 3.3.1. Sequencing, De Novo Assembly and Data Quality Control

Transcriptome sequencing was performed on 45 silique samples covering CK, SA, and IAA treatments. A total of 2,129,332,170 clean reads were generated. The Q30 base ratio exceeded 96.48%, the Q20 value was higher than 98.91%, and the GC content was above 45.92%, confirming the high quality of the sequencing data. Because of the absence of a publicly available reference genome for *I. indigotica*, the Trinity (v2.11.0) software was used for de novo assembly to generate unigenes as the reference transcripts. The mapping rate of clean reads was greater than 70.93%.

To obtain comprehensive functional gene information, unigenes were annotated against six mainstream databases, including GO, KEGG, EggNOG, NR, Swiss-Prot, and Pfam (Figure 4). In total, 30,323 unigenes were successfully functionally annotated. The NR database presented the highest annotation proportion (95.05%), followed by EggNOG (91.03%), GO (82.16%), Swiss-Prot (77.51%), Pfam (77.06%), and KEGG (39.35%). Detailed annotation statistics are summarized in Figure 3.

#### 3.3.2. Screening of Intra-Treatment Differentially Expressed Genes

Differentially expressed genes (DEGs) were screened based on the thresholds |log_2_FC| ≥ 2 and *p* < 0.05. The largest number of DEGs was identified in the T5-vs.-T4 comparison for all three treatments, indicating that the most dramatic transcriptional reprogramming occurs during the transition from silique browning maturation to full maturation. Pairwise intra-treatment comparisons were conducted for DEG identification, including CK2-vs.-CK1, CK3-vs.-CK2, CK4-vs.-CK3, CK5-vs.-CK4, IAA2-vs.-IAA1, IAA3-vs.-IAA2, IAA4-vs.-IAA3, IAA5-vs.-IAA4, SA2-vs.-SA1, SA3-vs.-SA2, SA4-vs.-SA3, and SA5-vs.-SA4.

Within the CK group, 37 DEGs were continuously upregulated and 1 DEG (*Iin05672*) was continuously downregulated across the first four developmental stages. Three DEGs (*Iin02938*, *Iin21547*, *Iin26253*) exhibited persistent upregulation throughout all five developmental stages (Figure 5). For the IAA-treated group, two DEGs (*Iin22730*, *Iin06713*) were continuously upregulated in the first four stages, while one DEG (*Iin06713*) maintained persistent upregulation across all five stages. Under SA treatment, nine DEGs showed sustained upregulation; two DEGs (*Iin21805*, *Iin26906*) displayed continuous downregulation during the first four stages; and four DEGs (*Iin21547*, *Iin11163*, *Iin11953*, *Iin02444*) were persistently upregulated throughout all five developmental stages.

##### EggNOG Functional Annotation of Intra-Treatment DEGs

DEGs derived from intra-treatment pairwise comparisons of the CK, IAA, and SA groups were aligned and functionally clustered using the EggNOG database. Although all three treatments shared identical protein functional categories, the number of annotated unigenes in each category varied significantly among groups. The four most enriched functional categories were post-translational modification, protein turnover and chaperones, signal transduction mechanisms, and carbohydrate transport and metabolism.

##### GO Functional Annotation of Intra-Treatment DEGs

GO functional annotation was performed for DEGs obtained from intra-group pairwise comparisons. The results revealed consistent primary GO term categories across all comparison groups, with differences only in the number of enriched DEGs per term. In the biological process (BP) category, the most abundant term was cellular process (GO:0009987), followed by metabolic process (GO:0008152), response to stimulus (GO:0050896), and biological regulation (GO:0065007). For the cellular component (CC) category, DEGs were predominantly enriched in cellular anatomical entity (GO:0110165) and protein-containing complex (GO:0032991). In the molecular function (MF) category, binding (GO:0005488) and catalytic activity (GO:0003824) had the largest number of enriched genes, followed by transcription regulator activity (GO:0140110) and transporter activity (GO:0005215). These results indicated that the transcriptional perturbations induced by CK, IAA, and SA treatments during *I. indigotica* silique development mainly occurred at the biological process and molecular function levels.

Further analysis of significantly enriched GO terms filtered by Q-value thresholds showed distinct treatment-specific enrichment patterns. In the CK group, DEGs were significantly enriched in BP terms, including carbohydrate metabolic process (GO:0005975), response to oxygen-containing compound (GO:1901700), and response to chemical (GO:0042221). At the MF level, significantly enriched terms included hydrolase activity acting on glycosyl bonds (GO:0016798), structural constituent of ribosome (GO:0003735), oxidoreductase activity (GO:0016491), and cytoskeletal protein binding (GO:0008092).

For the IAA-treated group, significantly enriched BP terms comprised diterpenoid biosynthetic process (GO:0016102), microtubule-based movement (GO:0007018), response to oxygen-containing compound (GO:1901700), and response to abiotic stimulus (GO:0009628). Major enriched MF terms included terpene synthase activity (GO:0010333), DNA-binding transcription factor activity (GO:0003700), and oxidoreductase activity (GO:0016491).

In the SA-treated group, DEGs were significantly enriched in BP terms such as polysaccharide metabolic process (GO:0005976), response to oxygen-containing compound (GO:1901700), secondary metabolic process (GO:0019748), and biosynthetic process (GO:0009058). Corresponding enriched MF terms included DNA-binding transcription factor activity (GO:0003700), oxidoreductase activity (GO:0016491), and mRNA binding (GO:0003729). The terms response to oxygen-containing compound (GO:1901700) and oxidoreductase activity (GO:0016491) were commonly enriched in all three groups, while the unique enriched pathways of IAA and SA treatments reflected the specific transcriptional responses of *I. indigotica* siliques to exogenous hormone stimuli.

##### KEGG Annotation of Intra-Treatment DEGs

Intra-treatment DEGs were annotated against the KEGG database. All groups shared the most dominant KEGG terms, with variations in the number of enriched DEGs. Obvious differences among the CK, IAA, and SA groups were observed in the metabolism category. In the CK group, carbohydrate metabolism and amino acid metabolism were the top two enriched pathways with the highest DEG abundance. For IAA treatment, terpenoid and polyketide metabolism, carbohydrate metabolism, secondary metabolite biosynthesis, and amino acid metabolism were the most enriched pathways. For SA treatment, carbohydrate metabolism, amino acid metabolism, and secondary metabolite biosynthesis constituted the predominant pathways.

Other functional categories exhibited consistent annotated terms across the three groups with quantitative differences in DEG enrichment. In the genetic information processing category, the top pathways included protein folding, sorting and degradation, DNA repair, and transcription. For the environmental information processing category, signal transduction and membrane transport were the most enriched pathways. Cellular processes were mainly enriched in transport and catabolism, while organismal systems were dominated by environmental adaptation pathways.

#### 3.3.3. Screening of Inter-Treatment Differentially Expressed Genes

DEGs were screened using the thresholds |log_2_FC| ≥ 2 and *p* < 0.05 for inter-treatment comparisons, including IAA-vs.-CK, SA-vs.-CK, and SA-vs.-IAA (Figure 6). The overlapping DEGs between SA-vs.-CK and IAA-vs.-CK represented genes modulated by exogenous SA and IAA relative to the control. DEGs identified from SA-vs.-IAA represented genes differentially regulated by SA compared with IAA. The intersection of these two DEG sets was defined as SA-responsive genes involved in *I. indigotica* silique development, including one upregulated transcript (Iin28812) and two downregulated transcripts (Iin19236, Iin14377). Similarly, IAA-responsive genes were identified from the intersection of common DEGs in IAA-vs.-CK/SA-vs.-CK and DEGs in IAA-vs.-SA comparisons, yielding 15 upregulated IAA-responsive candidate genes.

Inter-treatment DEGs at each developmental stage were further screened with the same thresholds. Stage-specific SA- and IAA-responsive genes were identified via pairwise intersection analysis for each time point. In total, 297 SA-responsive genes and 121 IAA-responsive genes were obtained across all five developmental stages, representing the core gene sets modulated by exogenous SA and IAA during *I. indigotica* silique growth and development.

##### EggNOG Annotation of Inter-Treatment DEGs

DEGs from IAA-vs.-CK, SA-vs.-CK, and SA-vs.-IAA comparisons were functionally clustered via EggNOG annotation (Figure 7). For IAA-vs.-CK DEGs, the signal transduction mechanism category contained the largest number of genes (126), followed by transcription (104) and post-translational modification, protein turnover and chaperones (96). For SA-vs.-CK DEGs, the transcription category had the maximum DEG count (163), followed by post-translational modification-related functions (124) and signal transduction mechanisms (108). For SA-vs.-IAA DEGs, post-translational modification, protein turnover, and chaperones were the most abundant category (167), followed by transcription (161) and signal transduction mechanisms (143). Collectively, exogenous SA and IAA primarily modulated core biological functions, including transcription, signal transduction, and post-translational modification, during *I. indigotica* silique development.

##### GO Annotation of Inter-Treatment DEGs

Inter-treatment DEGs were annotated using the GO database (Figure 8). All comparison groups shared the most enriched GO terms with variable DEG abundances. In the BP category, cellular process (GO:0009987), metabolic process (GO:0008152), and response to stimulus (GO:0050896) were dominant. In the CC category, cellular anatomical entity (GO:0110165) and protein-containing complex (GO:0032991) were the most enriched terms. In the MF category, binding (GO:0005488), catalytic activity (GO:0003824), and transcription regulator activity (GO:0140110) were predominant. Overall, the SA-vs.-CK comparison yielded more DEGs enriched in BP and MF terms than the IAA-vs.-CK comparison.

Further enrichment analysis revealed treatment-specific functional patterns (Figure 9). IAA-vs.-CK DEGs were significantly enriched in BP terms, including response to bacterium (GO:0009617) and response to other organism (GO:0051707), with no significant enrichment detected in CC and MF categories. SA-vs.-CK DEGs were significantly enriched in the BP terms response to oxygen-containing compound (GO:1901700) and response to chemical (GO:0042221); significantly enriched CC terms included DNA packaging complex (GO:0044815) and nucleosome (GO:0000786), while MF terms contained DNA-binding transcription factor activity (GO:0003700) and structural constituent of ribosome (GO:0030527).

##### KEGG Annotation of Inter-Treatment DEGs

Among 3782 inter-treatment DEGs, 1479 were successfully annotated to KEGG pathways. The MYB plant transcription factor pathway (K09422) contained the largest number of DEGs (27), followed by peroxidase (K00430, 20 DEGs), glutathione S-transferase (K00799, 14 DEGs), EREBP (K09286, 14 DEGs), and homeobox-leucine zipper protein (K09338, 14 DEGs). The auxin-related pathway (K09286; map04075) was also annotated, covering DEGs *Iin09664*, *Iin15103*, *Iin00972*, *Iin12397*, and *Iin09613*. KEGG annotation indicated that these DEGs were mainly involved in amino acid metabolism, oxidative phosphorylation, and transcriptional regulation pathways.

#### 3.3.4. Analysis of Differential-Responsive Genes

Core hormone-responsive genes were screened via intersection analysis of intra-treatment and inter-treatment DEGs (Figure 10). Nine SA-responsive candidate genes (*Iin21547*, *Iin23677*, *Iin25653*, *Iin09862*, *Iin02937*, *Iin22531*, *Iin06341*, *Iin08526*, *Iin26253*) and one unique IAA-responsive candidate gene (Iin22730) were identified.

A heatmap was constructed to visualize the dynamic expression profiles of 43 key genes, including the screened SA/IAA-responsive genes, 38 CK-specific developmental genes, and IAA pathway-related genes from KEGG annotation (Figure 11). All 43 genes were expressed in the CK, IAA, and SA groups with distinct stage-specific expression patterns. Genes in the CK and IAA groups showed high transcript abundance at T4 and T5, while SA-treated samples exhibited obvious expression signals at T3, T4, and T5.

GO annotation of the 43 core genes revealed diverse functional roles during silique development. *Iin06341* was annotated with 11 GO terms related to membrane components, ion transmembrane transport, and intracellular pH homeostasis. *Iin22531* was associated with lipid storage, reproductive development, and seed germination. Genes *Iin20493* and *Iin05738* were involved in DNA repair and protein ADP-ribosylation. *Iin20098* participated in lipid storage and reproductive processes. Notably, *Iin00972* was functionally linked to auxin response, auxin-activated signaling pathways, and sequence-specific DNA transcriptional regulation.

KEGG annotation showed that 14 of the 43 genes were mapped to nine functional pathways. In addition to five IAA pathway-related genes, *Iin20493* and *Iin05738* were enriched in the base excision repair pathway (K10798; map03410). *Iin02207*, *Iin26253*, and *Iin19473* were involved in vesicular transport, endoplasmic reticulum protein processing, and RNA degradation pathways, respectively.

#### 3.3.5. qRT-PCR Validation Analysis

Five candidate transcripts (*Iin05672*, *Iin26253*, *Iin09862*, *Iin05738*, *Iin00972*) were selected for qRT-PCR validation. All samples exhibited typical exponential amplification curves with Ct values ranging from 15.66 to 25.69, indicating reliable template quality and specific amplification. The relative expression levels calculated via the 2^−ΔΔCT^ method showed highly consistent expression trends between the transcriptome sequencing and qRT-PCR results (Figure 12), verifying the credibility and reproducibility of the transcriptomic data.

### 3.4. Metabolomic Profiling of Isatis indigotica Siliques

#### 3.4.1. Quality Control and Statistical Analysis of Metabolomic Samples

Pearson correlation analysis of quality control (QC) samples was performed to evaluate metabolomic data stability. All correlation coefficients of QC samples exceeded 0.99 throughout the detection process, demonstrating high stability and reproducibility of the mass spectrometry detection system.

Principal component analysis (PCA) revealed tight clustering of biological replicates and good sample correlation. PC1 clearly separated samples from different developmental stages, while PC2 distinguished samples from different hormone treatments. Orthogonal partial least-squares discriminant analysis (OPLS-DA) showed clear inter-group separation, and all Q^2^ values of OPLS-DA models were greater than 0.5, indicating robust model fitness and reliable variable importance in projection (VIP) values for differential metabolite screening.

A total of 7028 metabolites were annotated across 90 samples, including 4961 metabolites detected in positive ion (POS) mode and 2067 in negative ion (NEG) mode. Metabolite classification analysis identified 21 categories in both ion modes. In POS mode, amino acids and their derivatives were the dominant category (30.69%, 1522 metabolites), followed by benzene and substituted derivatives (11.30%, 560 metabolites) and organic acids (10.54%, 523 metabolites). In NEG mode, organic acids were the most abundant class (22.59%, 467 metabolites), followed by benzene and substituted derivatives (11.03%, 228 metabolites) and amino acids and their derivatives (9.70%, 200 metabolites) (Figure 13).

#### 3.4.2. Screening of Intra-Treatment Differential Accumulated Metabolites

Differentially accumulated metabolites (DAMs) were screened based on the thresholds |log_2_FC| ≥ 1 and VIP > 1 for intra-treatment pairwise comparisons. For the CK group (Figure 14), the T5-vs.-T4 comparison produced the largest number of DAMs (2920), including 1124 upregulated and 1796 downregulated metabolites. One DAM (MW0123498) was continuously upregulated, and one DAM (MW0154300) was continuously downregulated across the first four stages, with 681 stage-specific upregulated DAMs identified at T5.

For the IAA-treated group (Figure 15), the total DAM number increased gradually with silique development, with the maximum value (2834 DAMs) observed in the T5-vs.-T4 comparison (1024 upregulated, 1810 downregulated). Ten DAMs were continuously downregulated across the first four stages, and one DAM (MW0016076) maintained persistent downregulation throughout all five stages, with 732 T5-specific upregulated DAMs.

For the SA-treated group (Figure 15), the T3-vs.-T2 comparison yielded the highest DAM abundance (2696 DAMs; 1288 upregulated, 1408 downregulated). Eleven DAMs were continuously upregulated, and four DAMs were persistently downregulated during the first four stages, among which MW0152923 was downregulated across all five stages, with 582 T5-specific upregulated DAMs. Overall, the CK and IAA groups exhibited the most dramatic metabolic changes at T5, while the SA group showed the strongest metabolic perturbation at the T3 expansion stage.

#### 3.4.3. Screening of Inter-Treatment Differential Accumulated Metabolites

Inter-treatment DAM screening identified 1351 DAMs for IAA-vs.-CK (595 upregulated, 756 downregulated), 1617 DAMs for SA-vs.-CK (635 upregulated, 982 downregulated), and 856 DAMs for SA-vs.-IAA (386 upregulated, 470 downregulated). Via intersection analysis, 61 SA-responsive metabolites (17 upregulated, 44 downregulated) and 22 IAA-responsive metabolites (4 upregulated, 18 downregulated) were identified as core hormone-modulated metabolites during silique development.

Stage-specific responsive metabolites were further screened for each developmental time point. At T1, 332 SA-responsive metabolites (182 upregulated, 150 downregulated) and 54 IAA-responsive metabolites (15 upregulated, 39 downregulated) were obtained. At T2, 414 SA-responsive metabolites and 156 IAA-responsive metabolites were identified. At T3, 234 SA-responsive metabolites and 101 IAA-responsive metabolites were screened. At T4, 68 SA-responsive metabolites and 37 IAA-responsive metabolites were detected. At T5, 112 SA-responsive metabolites and 82 IAA-responsive metabolites were retrieved.

#### 3.4.4. Differential-Responsive Metabolites to Exogenous IAA and SA

Cumulative analysis across all developmental stages identified 392 IAA-responsive metabolites (146 upregulated, 254 downregulated) and 965 SA-responsive metabolites (491 upregulated, 491 downregulated). Further screening obtained 308 IAA-specific DAMs and 881 SA-specific DAMs. Among them (Figure 16), 12 metabolites were persistently modulated by IAA throughout silique development, and 36 metabolites showed consistent responses to SA across all developmental stages.

Considering the critical role of mature silique metabolism in seed quality, T5-stage DAMs were specifically analyzed (Figure 17). A total of 341, 401, and 548 stage-specific DAMs were identified in the CK, IAA, and SA groups, respectively, at the full maturation stage.

#### 3.4.5. KEGG Functional Annotation and Enrichment Analysis of DAMs

##### Functional Analysis of Inter-Treatment DAMs

KEGG annotation of core hormone-responsive DAMs showed distinct metabolic patterns between IAA and SA treatments. Among 308 IAA-responsive DAMs, 29 were annotated to functional pathways, dominated by carbohydrate metabolism (19 metabolites), followed by cofactor biosynthesis, nucleotide metabolism, and secondary metabolite biosynthesis. Among 881 SA-responsive DAMs, 92 were functionally annotated, with carbohydrate metabolism (59 metabolites) as the primary pathway, followed by amino acid biosynthesis, nucleotide metabolism, ABC transporters, and cofactor biosynthesis.

##### Functional Analysis of T5-Stage DAMs

T5-stage DAM annotation revealed stage-specific metabolic characteristics. In the SA group, 65 annotated DAMs were mainly enriched in carbohydrate metabolism (47 metabolites), carbon metabolism, cofactor biosynthesis, and secondary metabolite biosynthesis. In the IAA group, 29 annotated DAMs were primarily involved in carbohydrate metabolism (18 metabolites), tryptophan metabolism, and cofactor biosynthesis. In the CK group, 25 annotated DAMs were dominated by carbohydrate metabolism (21 metabolites), together with arginine and proline metabolism and secondary metabolite biosynthesis. Collectively, carbohydrate metabolism represented the core metabolic pathway regulating silique maturation and seed quality across all treatments, with exogenous SA and IAA inducing specific metabolic perturbations.

### 3.5. Integrated Transcriptome–Metabolome Analysis

Nine-quadrant correlation analysis was performed using 43 core differential genes, 36 SA-responsive metabolites, and 12 IAA-responsive metabolites to reveal key gene–metabolite regulatory modules. Quadrants 3 and 7 represent gene–metabolite pairs with consistent expression and accumulation trends, while Quadrants 1 and 9 represent pairs with opposite variation patterns.

For SA-responsive gene–metabolite pairs (Figure 18), all SA-modulated metabolites were significantly correlated with 15 core SA-responsive genes. Genes *Iin05738*, *Iin08526*, and *Iin10645* exhibited opposite variation trends with multiple metabolites in Quadrant 9, while metabolite MW0164988 showed inverse accumulation patterns with 12 core genes in Quadrant 1.

For IAA-responsive gene–metabolite pairs, all IAA-modulated metabolites were significantly correlated with 12 core IAA-responsive genes. Eight genes showed negative correlations with multiple metabolites in Quadrant 9, and four genes exhibited inverse variation trends with a series of metabolites in Quadrant 1.

KEGG pathway analysis showed that IAA treatment mainly activated terpenoid and polyketide metabolism, carbohydrate metabolism, secondary metabolite biosynthesis, and amino acid metabolism. SA treatment predominantly regulated carbohydrate metabolism, amino acid metabolism, and secondary metabolite biosynthesis. Further screening revealed the following key functional genes driving hormone-specific metabolic regulation: *Iin05738*, *Iin09862*, and *Iin05672* for SA treatment, and *Iin15103*, *Iin02207*, *Iin26253*, *Iin22733*, *Iin12397*, *Iin09613*, *Iin00972*, and *Iin09664* for IAA treatment.

## 4. Discussion

### 4.1. Regulation of Carbohydrate Metabolism in Isatis indigotica Siliques by Exogenous SA and IAA

Carbohydrates act not only as structural substrates for silique morphogenesis, but also as core carbon skeletons and energy donors for the biosynthesis of seed storage compounds. Hormonal signals mediate the dynamic reprogramming of carbohydrate synthesis, degradation, and translocation by regulating the transcription and enzymatic activity of key sugar metabolism enzymes, thereby determining the growth rate and developmental fate of plant reproductive organs [20]. The physiological data obtained in this study confirmed that exogenous SA application at the full-bloom stage significantly promoted starch accumulation during silique expansion (T2–T3 stages). Meanwhile, SA treatment maintained relatively high activities of critical carbohydrate metabolism enzymes, including invertase, glucose-6-phosphate dehydrogenase (G6PDH), and pyruvate kinase (PK), which accelerated metabolic processes in early-stage siliques.

As a rate-limiting enzyme of the oxidative pentose phosphate pathway, G6PDH provides reducing power and pentose precursors for the biosynthesis of storage compounds and plays an essential role in the development of reproductive organs such as seeds and siliques [21]. In the present study, G6PDH activity in SA-treated siliques was significantly higher than that in the control (CK) group across the T1–T4 stages, indicating that appropriate exogenous SA supplementation sustains high metabolic activity in silique tissues. This provides sufficient material prerequisites for cell expansion and storage substance accumulation, consequently promoting the rapid development of siliques.

Siliques of Brassicaceae species are composite organs consisting of seeds encapsulated by the silique wall (pericarp). At the fully mature T5 stage, the silique wall undergoes progressive senescence and programmed cell death. Accordingly, enzyme activity and metabolite profiles measured at this stage represent mixed signals derived from both senescent silique walls and developing seeds, and thus cannot clearly reflect the overall physiological activity of intact silique tissues. This phenomenon is a common technical limitation in physiological studies of Brassicaceae silique material [22].

Transcriptomic results revealed that high-concentration IAA treatment applied at the flower-bud stage significantly enriched the diterpenoid biosynthesis pathway in *I. indigotica* siliques. The diterpenoid metabolic branch generates not only growth-regulating hormone precursors (e.g., gibberellins) but also a variety of defensive secondary metabolites. Sustained overactivation of defensive metabolic pathways consumes substantial cellular carbon sources, reducing equivalents, and energy, which compete for metabolic resources with reproductive organ growth and ultimately disrupt normal developmental progression [23]. Consistent with the inhibitory effect of high-concentration IAA on *I. indigotica* silique development observed herein, a previous study on peach fruit expansion reported that excessive exogenous auxin induces the upregulation of defense-related transcriptional modules and represses normal fruit growth [24].

A canonical growth–defense trade-off is prevalent in plants, whereby exogenous signal perturbations remodel carbon partitioning between growth-related metabolic pathways and defensive secondary metabolism [25]. Combined with phenotypic data acquired from identical treatments in our published work (https://doi.org/10.13422/j.cnki.syfjx.20260416), we hypothesize that high-concentration IAA application at the flower-bud stage excessively activates secondary metabolic defense pathways in siliques and reshuffles carbon resource allocation, which may disturb the normal developmental rhythm of *I. indigotica* siliques. It should be noted that macroscopic phenotypic traits were not re-measured in the current study, as these data have been published elsewhere. Our interpretation is built upon integrating the physiological and multi-omics data generated in the present study together with the previously reported phenotypic dataset. This hypothesis is further supported by preliminary seed germination assays: seeds harvested from the IAA-treated group exhibited a markedly lower germination rate than those from the CK group (47.00% for IAA vs. 88.67% for CK).

### 4.2. SA- and IAA-Mediated Transcriptional Reprogramming in Isatis indigotica Siliques

Exogenous hormone-modulated reproductive organ development is a sophisticated biological process coordinated by multiple genes and dynamically interacting pathways. Temporal transcriptomic reprogramming across successive developmental stages constitutes the molecular basis by which plants perceive exogenous hormonal cues and reshape organ growth trajectories [26,27]. As core endogenous signaling molecules, SA and IAA regulate reproductive development in a dose- and application-time-dependent manner: physiologically optimal concentrations modulate normal developmental programs, whereas excessive concentrations or inappropriate application timings preferentially activate stress and defense transcriptional modules, thereby perturbing regular reproductive development [28,29]. In citrus fruit ripening, for instance, hormonal signals drive global transcriptional reprogramming of cell wall metabolism, secondary metabolism, and hormone signal transduction modules to govern fruit maturation [30].

GO enrichment analysis in this study demonstrated that response to oxygen-containing compounds and oxidoreductase activity were core responsive pathways shared by the CK and hormone-treated groups. This finding supports the conclusion that redox homeostasis serves as the fundamental molecular basis for sustaining normal silique growth and development in *I. indigotica* [31]. Exogenous SA treatment significantly enriched terms associated with polysaccharide metabolism, mRNA binding, and DNA-binding transcription factor activity, suggesting that SA activates polysaccharide and oxygen-containing compound metabolic networks via hierarchical transcription factor regulation. Such activation provides sufficient substances and energy for cell proliferation and turnover, ultimately facilitating silique growth and expansion.

In contrast, IAA treatment induced prominent enrichment of diterpenoid biosynthesis, microtubule-based movement, and abiotic stimulus response pathways [32,33]. From a molecular regulatory perspective, exogenous IAA signals presumably activate microtubule-mediated intracellular signaling cascades through transcription factor regulatory hierarchies, initiate diterpenoid secondary metabolism programs, and consequently reshape silique developmental processes. Further KEGG annotation revealed divergent pathway preferences between SA and IAA treatments. The SA-treated group exhibited substantially higher enrichment of genes involved in carbohydrate and amino acid metabolism than the CK group. Previous studies on Brassica napus siliques have confirmed that SA signaling modulates the expression of amino acid transporter genes and mediates the translocation and reallocation of amino acids within siliques to support seed storage protein biosynthesis [34]. These published findings corroborate the SA-induced enhancement of amino acid metabolism observed in the present study.

Differentially expressed genes (DEGs) from the IAA treatment group were significantly enriched in terpenoid and polyketide metabolism pathways. Certain diterpenoid metabolic intermediates act as gibberellin biosynthesis precursors and participate in the morphogenetic regulation of plant reproductive organs [35,36]. The significant enrichment of carbohydrate metabolism pathways in the CK, SA, and IAA groups further indicates that sugar metabolic networks function as an indispensable core regulatory module for *I. indigotica* silique development [37,38].

Nine SA-responsive genes and one IAA-responsive candidate gene were identified via multi-omics intersection screening. Functional annotation based on the NR database revealed the potential roles of these core genes: *Iin21547* (Gene ID: 106376214) encodes a chloroplast envelope protein involved in metabolite exchange and signal communication between plastids and the cytosol [39]; *Iin06341* (Gene ID: 18017168) belongs to the Na^+^/H^+^ exchanger family and participates in cellular ion homeostasis and pH balance regulation [40,41]; *Iin20493* (Gene ID: 106408172) encodes a PARP-family protein that mediates DNA ADP-ribosylation and functions in DNA damage repair and cell fate determination [42]; *Iin20098* (Gene ID: 103850173) is an oleosin homolog that primarily regulates seed oil body assembly and lipid storage [43]; *Iin09862* (Gene ID: 108810508) is a TIP3-2 aquaporin homolog, which is presumed to modulate cellular turgor pressure to drive silique cell expansion. Notably, these functional interpretations are indirect inferences derived solely from homologous sequence annotation and have not been validated by in vivo or in vitro functional assays. The authentic biological functions of these genes during *I. indigotica* silique development remain to be further elucidated.

### 4.3. Physiological Roles of Differentially Accumulated Metabolites in Isatis indigotica Siliques Under Exogenous SA and IAA Treatments

Metabolites represent the ultimate phenotypic output of gene expression. Untargeted metabolomics enables the panoramic profiling of in vivo metabolite dynamics and helps uncover the intrinsic mechanisms underlying plant biological processes [44]. A total of 7028 metabolites were identified across positive ion (POS) and negative ion (NEG) detection modes in this study, mainly including amino acids and their derivatives, organic acids, and benzene and substituted derivatives. Amino acids not only serve as building blocks for protein biosynthesis but also act as signaling molecules modulating plant growth and stress responses. Plants remodel amino acid metabolism to adapt to external environmental changes and exogenous hormonal stimuli [45,46]. During legume pod development, amino acids are translocated between vegetative and reproductive organs to provide nitrogen sources for seed protein biosynthesis [47,48], and exogenous SA can reshape amino acid metabolic profiles under stress conditions [49]. In this study, both SA and IAA treatments significantly perturbed amino-acid-associated metabolic pathways, indicating that amino acid metabolism is a vital component of the hormonal response in *I. indigotica* siliques.

As primary photosynthetic products, carbohydrates provide energy and carbon skeletons for plant organ growth and directly affect seed development and reserve accumulation [50]. Multiple crop studies have confirmed that exogenous growth regulator application remodels carbohydrate metabolic networks, thereby altering the developmental trajectories of fruits and seeds [51]. In the present study, the SA-treated group accumulated far more differentially accumulated metabolites (DAMs) enriched in carbohydrate metabolism pathways than the IAA-treated group, which metabolically explains the phenotypic improvement of silique development following SA application at the full-bloom stage.

The T5 stage corresponds to full silique maturity and acts as a critical switch for substance accumulation and developmental transition. Our results demonstrated that the tryptophan, phenylalanine, and tyrosine metabolism pathways remained highly active in IAA-treated mature siliques. Tryptophan serves as a key precursor for auxin biosynthesis, while phenylalanine participates in phenylpropanoid metabolism and lignin synthesis [52,53]. We hypothesize that siliques treated with high-concentration IAA at the flower-bud stage maintain elevated secondary metabolic activity even at the full maturation stage, leading to delayed developmental transition. This inference is strongly supported by seed germination results: seeds from IAA-treated plants exhibited significantly lower vigor and germination rates than CK seeds (47.00% for IAA vs. 88.67% for CK). In contrast, carbon metabolism pathways were prominently enriched in SA-treated siliques at the T5 stage. Carbon metabolism provides carbon skeletons for amino acid and nucleic acid synthesis and participates in the late-stage modification of seed storage reserves [54,55], which facilitates seed maturation within siliques.

### 4.4. Multi-Omic Integration of Physiological, Transcriptomic, and Metabolomic Data to Dissect the Regulatory Network Underlying Exogenous SA- and IAA-Mediated Silique Development in Isatis indigotica

Single-omics approaches only capture biological alterations at an individual molecular layer, whereas integrated transcriptome–metabolome analysis enables the identification of correlated gene–metabolite modules and facilitates the dissection of regulatory cascades linking gene expression to metabolite accumulation [56,57]. In this study, nine-quadrant correlation analysis screened three core SA-responsive genes (*Iin05738*, *Iin09862*, *Iin05672*) and eight core IAA-responsive genes (*Iin15103*, *Iin02207*, *Iin26253*, *Iin22733*, *Iin12397*, *Iin09613*, *Iin00972*, *Iin09664*). Gene and protein functional information was retrieved from the NCBI database based on NR annotation accession IDs.

*Iin09862* (Gene ID: 108810508) encodes a TIP3-2 aquaporin homolog. It is speculated that SA upregulates this gene to modulate cellular turgor pressure and consequently promote silique cell expansion. Meanwhile, *Iin00972* encodes an auxin-responsive transcription factor that mediates the sensitivity of siliques to IAA signals. Based on the comprehensive analysis of physiological indices, transcriptomic, and metabolomic datasets, we proposed a hypothetical regulatory model illustrating the molecular mechanism by which exogenous SA and IAA differentially govern *I. indigotica* silique development (Figure 19).

Specifically, SA application at the full-bloom stage activates DNA-binding transcription factors and upregulates key genes involved in carbohydrate and amino acid metabolism. This transcriptional reprogramming increases the activities of metabolic enzymes (including invertase, G6PDH, and PK), promotes the accumulation of starch and amino-acid-derived metabolites, accelerates silique cell growth and substance filling, and ultimately exerts positive regulatory effects on silique development. In contrast, high-concentration IAA spraying at the flower-bud stage triggers extensive activation of secondary metabolic pathways (e.g., diterpenoid and tryptophan metabolism). This excessive activation consumes endogenous nutritional resources in siliques, disrupts the normal temporal progression of carbohydrate metabolism, delays silique developmental transition, and ultimately inhibits silique growth and development.

### 4.5. Limitations and Future Perspectives of the Present Study

This study has several inherent limitations. First, due to the lack of a high-quality reference genome for *I. indigotica*, transcriptome analysis relied on de novo assembly, which may limit the completeness of gene functional annotation. Second, only five representative developmental stages were sampled in this study; thus, subtle and transient developmental events throughout the entire silique ontogeny cannot be fully captured. Third, the current study only established correlative relationships between gene expression and phenotypic variation. Candidate responsive genes were screened merely based on transcriptomic expression patterns, without further in vivo and in vitro functional validation (e.g., VIGS-mediated gene silencing and heterologous transformation). Therefore, the authentic biological functions of core candidate genes (e.g., *Iin09862* and *Iin00972*) during *I. indigotica* silique development require further experimental verification. Fourth, macroscopic phenotypic traits, including silique size, seed-set rate, and seed yield under these exact hormone treatments, have been described in our previous publication (https://doi.org/10.13422/j.cnki.syfjx.20260416) and are not repeated in the present manuscript. The causal links between molecular physiological changes and developmental phenotypes are inferred by cross-referencing our multi-omics dataset with an independent phenotypic dataset. Further functional validation of candidate genes is still required to establish direct causal relationships.

Collectively, this study systematically elucidated the molecular mechanisms of exogenous SA- and IAA-regulated silique development in *I. indigotica* at the physiological, transcriptomic, and metabolomic levels. We confirmed that SA application at the full-bloom stage promotes silique development, whereas high-concentration IAA treatment at the flower-bud stage exerts inhibitory effects. These findings provide a theoretical basis for optimizing the application timing and concentration of plant growth regulators in the standardized seed propagation of *I. indigotica*. Furthermore, the SA- and IAA-responsive candidate genes identified in this work supply valuable genetic resources for the molecular breeding of this medicinal plant.

Future research will establish gradient hormone treatments with multiple concentrations and application timings to refine hormone-mediated regulatory patterns. Combined with systematic gene functional validation and seed vigor evaluation, these efforts are expected to help in further dissecting the precise molecular mechanisms underlying hormone-regulated silique development and seed formation in *I. indigotica*.

## 5. Conclusions

By integrating physiological parameter measurements with transcriptomic and metabolomic analyses, this study elucidated the physiological and molecular mechanisms through which exogenous SA and IAA regulate silique growth and development in *I. indigotica*. The results demonstrated that exogenous SA and IAA significantly reshape the accumulation profiles of key nutritional compounds, including sucrose, starch, and soluble proteins, while modulating the activities of critical carbohydrate-metabolizing enzymes, such as invertase, pyruvate kinase (PK), glucose-6-phosphate dehydrogenase (G6PDH), and soluble starch synthase (SSS). SA application at the full-bloom stage substantially promoted starch accumulation during silique expansion (T2–T3) and facilitated substance filling within silique tissues. In comparison, treatment with 0.571 mmol·L^−1^ IAA at the flower-bud stage remodeled the temporal dynamics of metabolic processes in siliques. These metabolic alterations correspond to the phenotypic performance documented in our published study (https://doi.org/10.13422/j.cnki.syfjx.20260416). Transcriptomic screening identified nine SA-responsive genes and one IAA-responsive candidate gene closely associated with silique development. GO and KEGG enrichment analyses revealed distinct regulatory patterns of the two phytohormones: SA primarily modulates carbohydrate and amino acid metabolism pathways, whereas IAA-induced molecular perturbations are mainly enriched in terpenoid and polyketide metabolism pathways. A total of 7028 metabolites were detected via untargeted metabolomics, predominantly comprising amino acids and their derivatives, organic acids, and benzene-related compounds. Notably, SA exerted a more pronounced regulatory effect on carbohydrate metabolism pathways than IAA. Numerous treatment-specific differentially accumulated metabolites were identified at the full silique maturity stage, indicating that exogenous hormonal treatments profoundly alter metabolite deposition during silique ripening. Furthermore, nine-quadrant integrated transcriptome–metabolome analysis pinpointed three core SA-responsive and eight core IAA-responsive genes, constructing a series of gene–metabolite correlation modules that are tightly linked to silique developmental regulation.

Collectively, this study characterizes the physiological and multi-omics molecular responses of *I. indigotica* siliques triggered by exogenous SA and IAA, thus providing a solid theoretical basis for the rational application of exogenous phytohormones in the standardized seed propagation of this medicinal species. The hormone-responsive candidate genes identified in this work offer valuable genetic resources for future mechanistic studies and molecular-breeding research on *I. indigotica*.

## Figures and Tables

**Figure 1:**
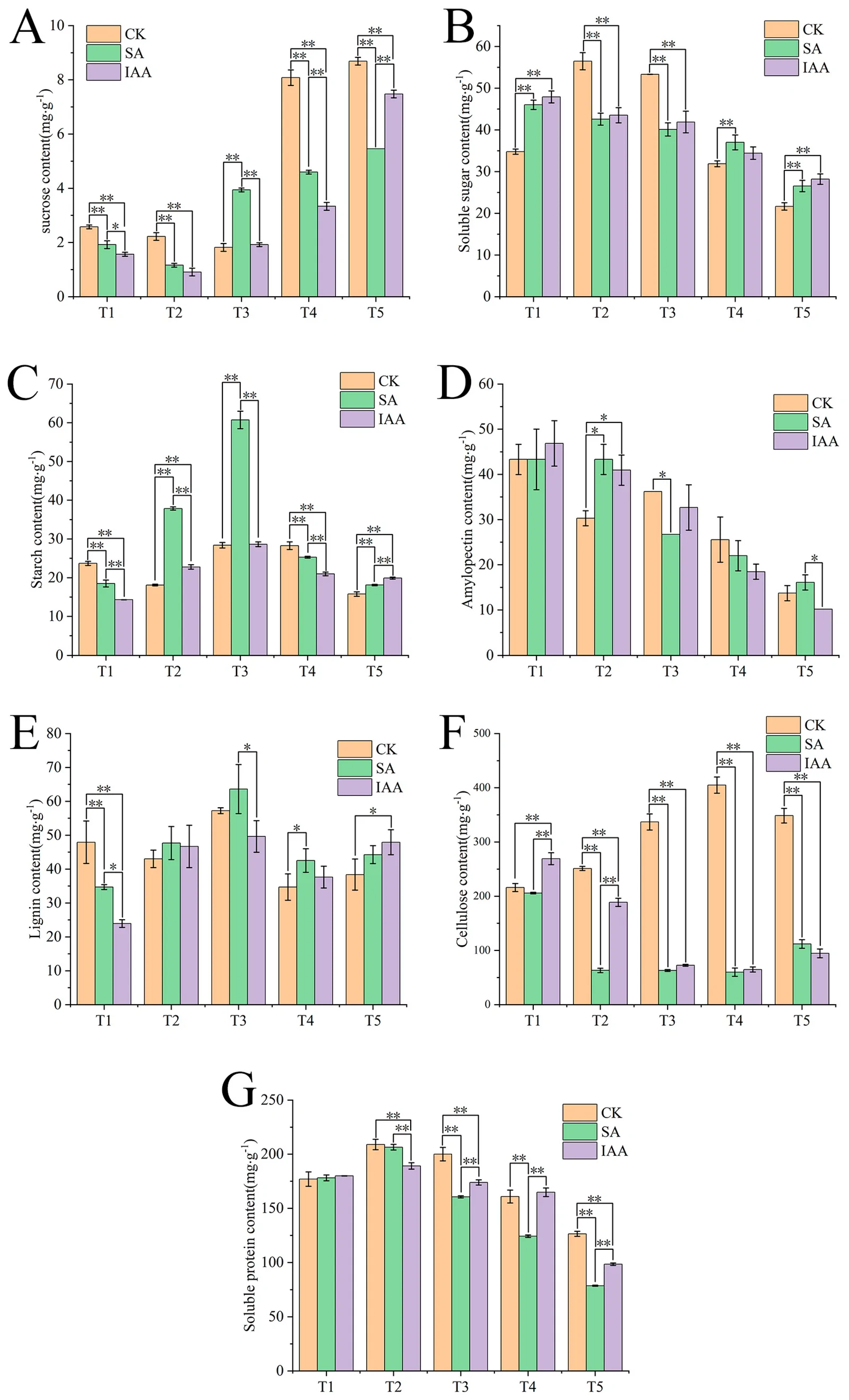
Nutrient content in siliques of *Isatis indigotica* under exogenous IAA and SA treatments: sucrose content (**A**), soluble sugar content (**B**), starch content (**C**), amylopectin content (**D**), lignin content (**E**), cellulose content (**F**), and soluble protein content (**G**). T1 represents the silique formation stage, T2 represents the silique growth stage, T3 represents the silique expansion stage, T4 represents the silique browning-maturation stage, and T5 represents the silique full-maturation stage. * denotes significant difference (*p* < 0.05); ** denotes very significant difference (*p* < 0.01).

**Figure 2:**
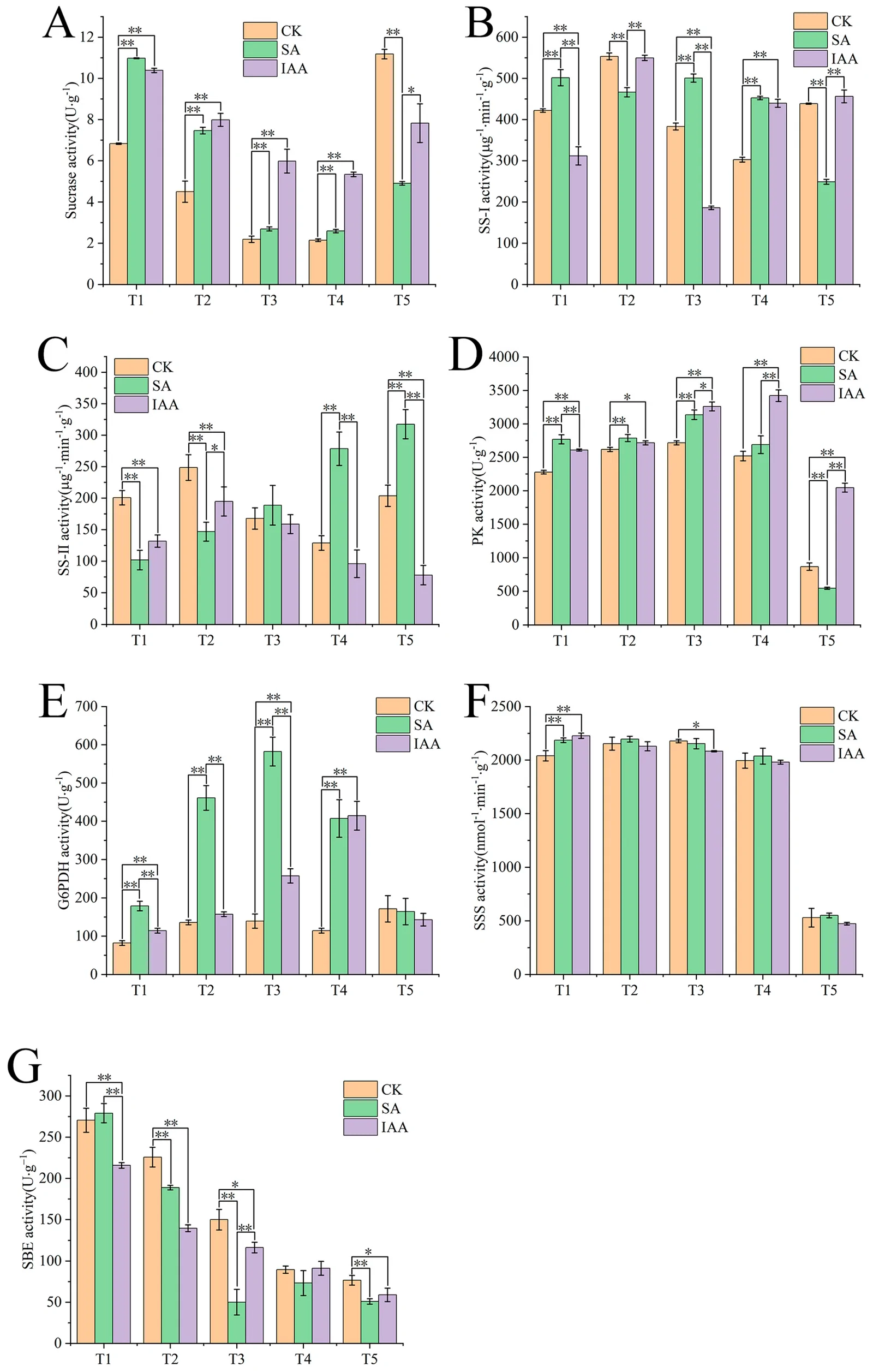
Changes in physiologically active substances in *Isatis indigotica* siliques under exogenous IAA and SA treatments: sucrase activity (**A**), sucrose synthase (cleavage direction, SS-I) activity (**B**), sucrose synthase (synthetic direction, SS-II) activity (**C**), pyruvate kinase activity (**D**), glucose-6-phosphate dehydrogenase activity (**E**), soluble starch synthase activity (**F**), and starch-branching enzyme activity (**G**). T1 represents the silique formation stage, T2 represents the silique growth stage, T3 represents the silique expansion stage, T4 represents the silique browning-maturation stage, and T5 represents the silique full-maturation stage. * denotes significant difference (*p* < 0.05); ** denotes very significant difference (*p* < 0.01).

**Figure 3:**
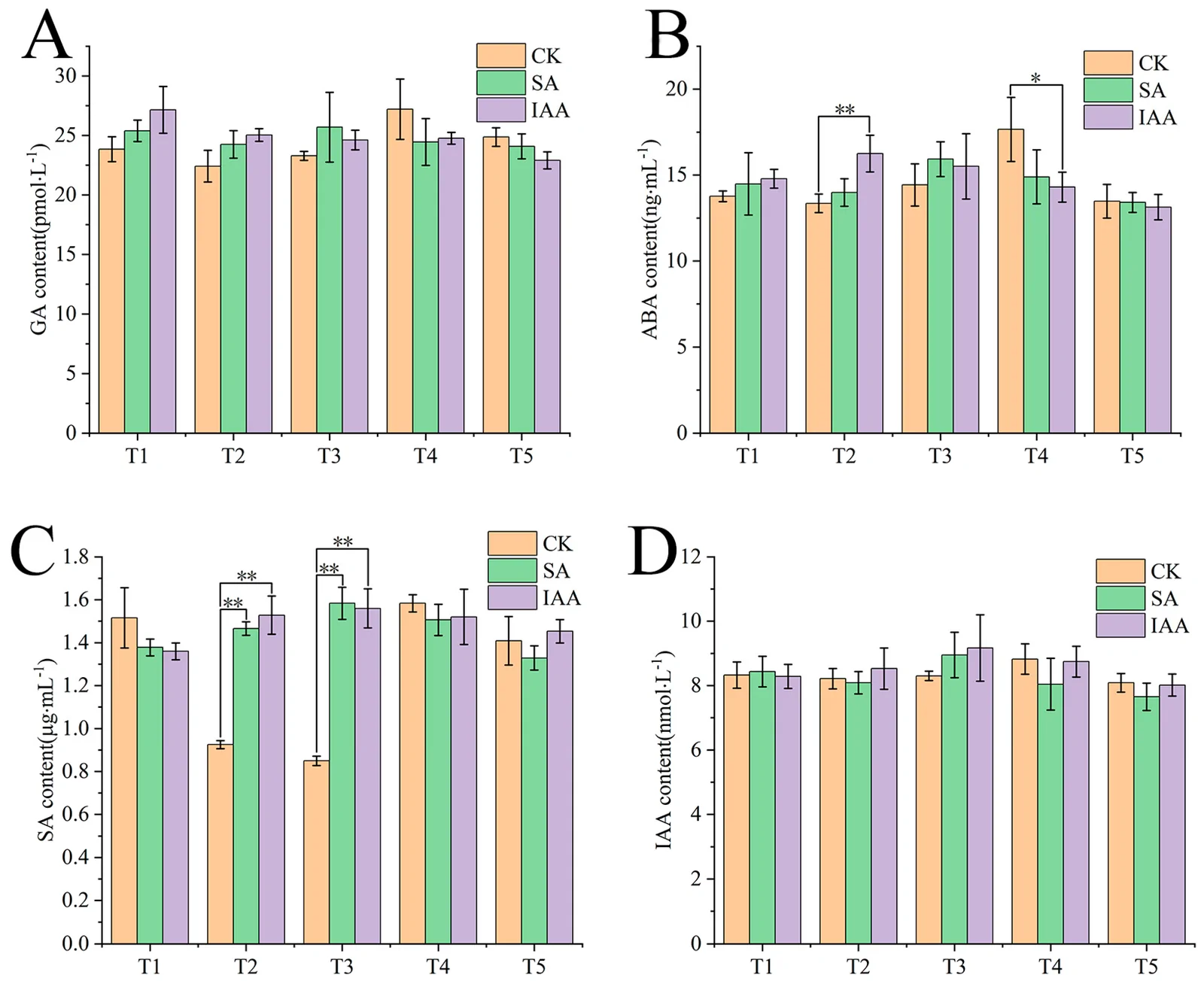
Contents of endogenous hormones in *Isatis indigotica* siliques under exogenous IAA and SA treatments: endogenous GA content (**A**), endogenous ABA content (**B**), endogenous SA content (**C**), and endogenous IAA content (**D**). T1 represents the silique formation stage, T2 represents the silique growth stage, T3 represents the silique expansion stage, T4 represents the silique browning-maturation stage, and T5 represents the silique full-maturation stage. * denotes significant difference (*p* < 0.05); ** denotes very significant difference (*p* < 0.01).

**Figure 4:**
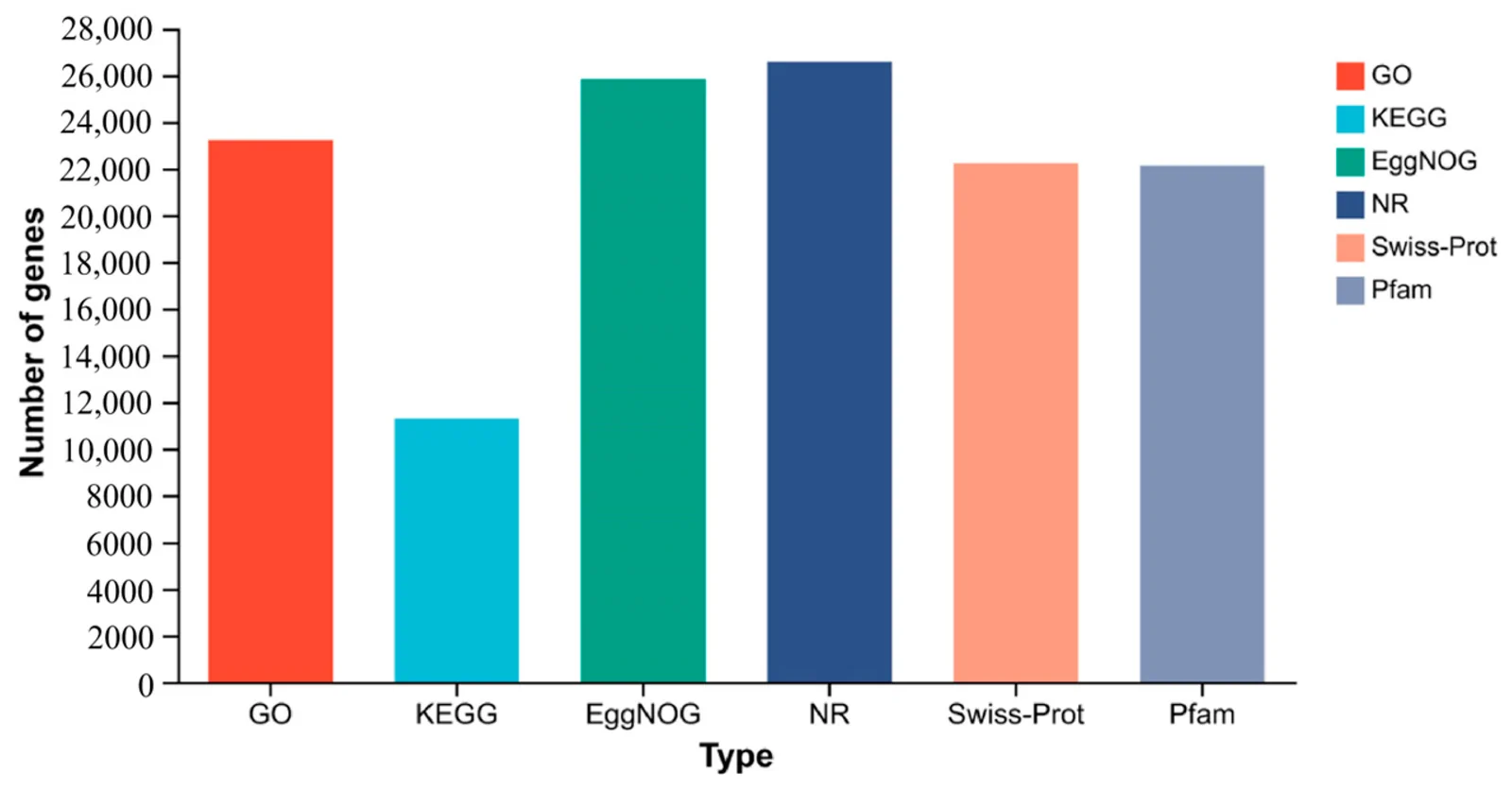
Functional annotation statistics of the Unigene database.

**Figure 5:**
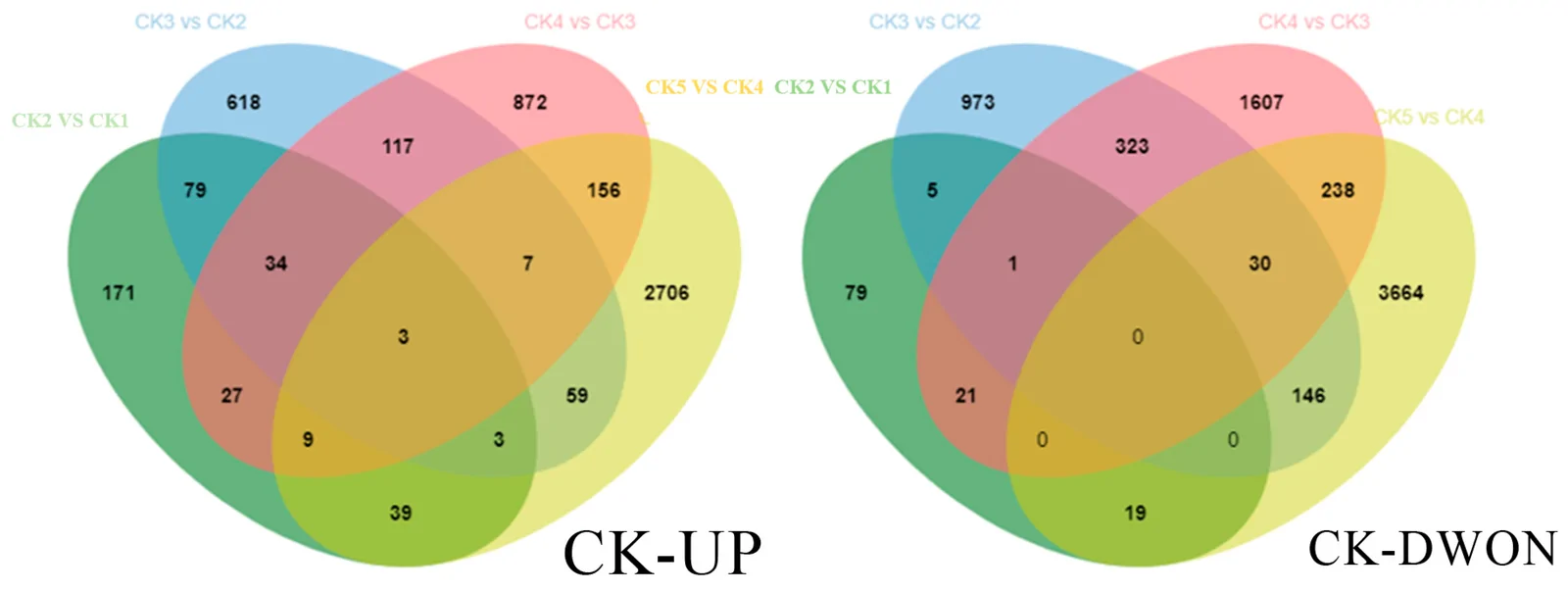

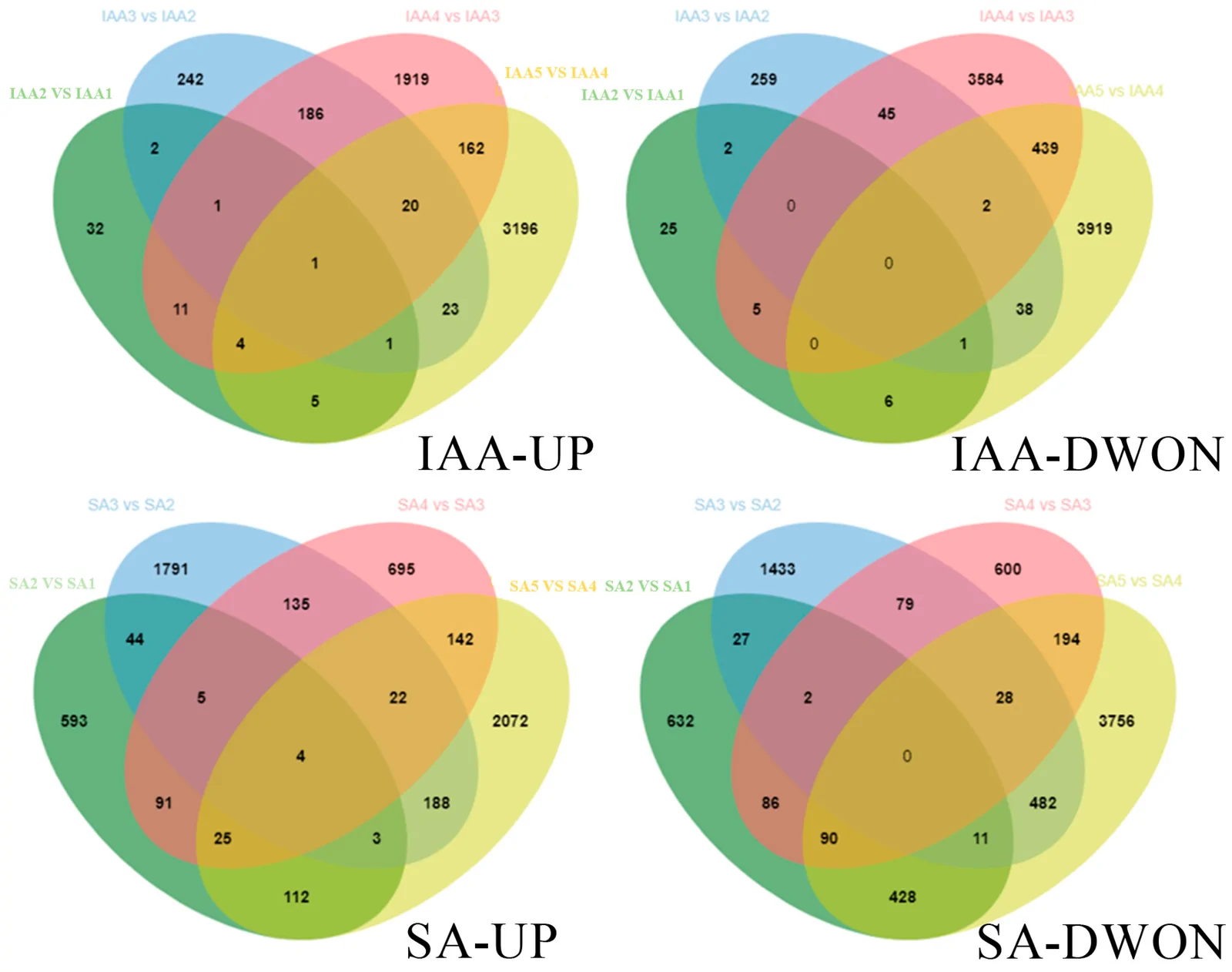
Screening results of intra-group differentially expressed genes.

**Figure 6:**
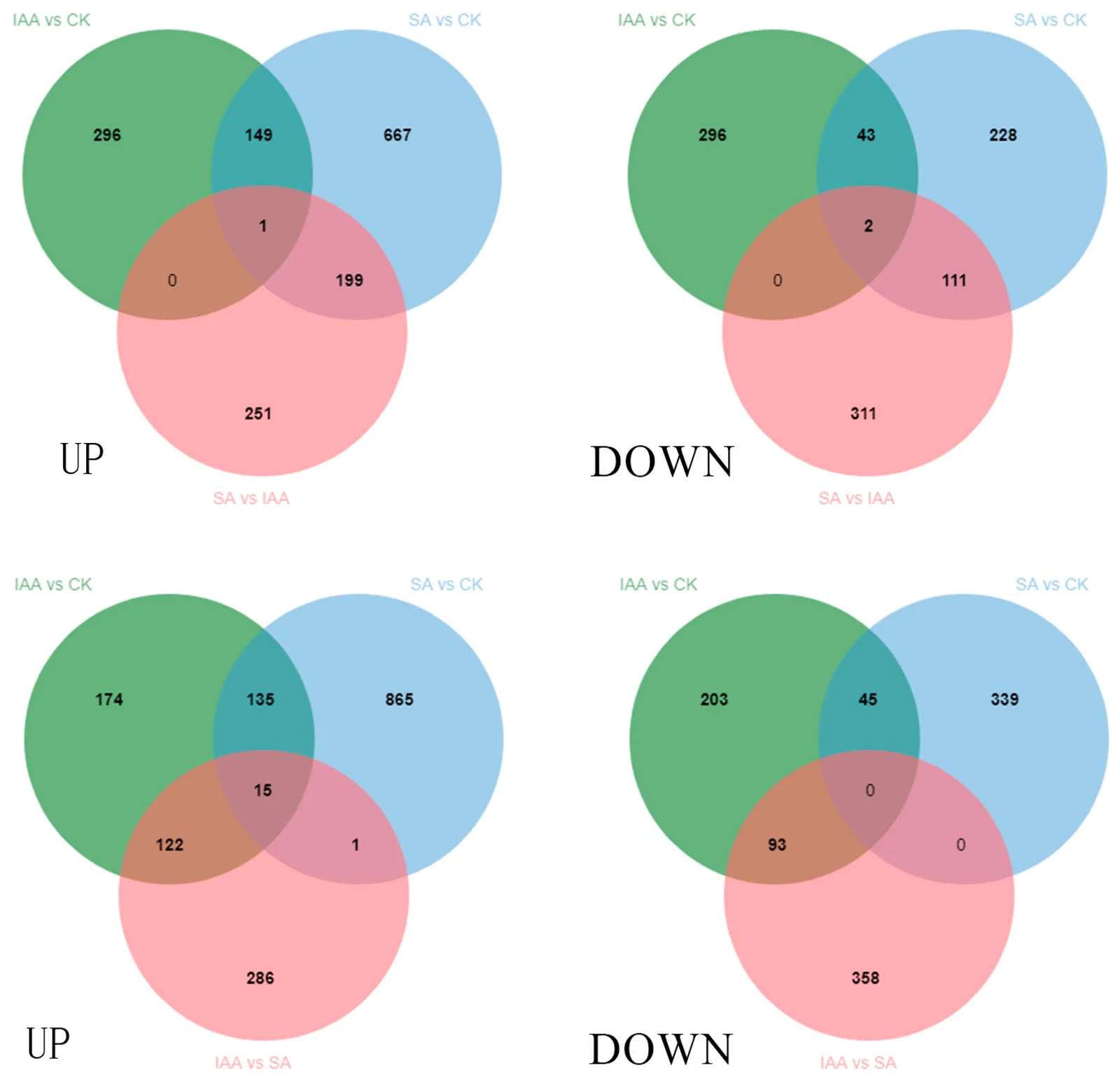
Responsive genes involved in silique growth and development under inter-group comparisons of exogenous SA and IAA treatments. (**left**) panel: genes responsive to exogenous SA; (**right**) panel: genes responsive to exogenous IAA.

**Figure 7:**
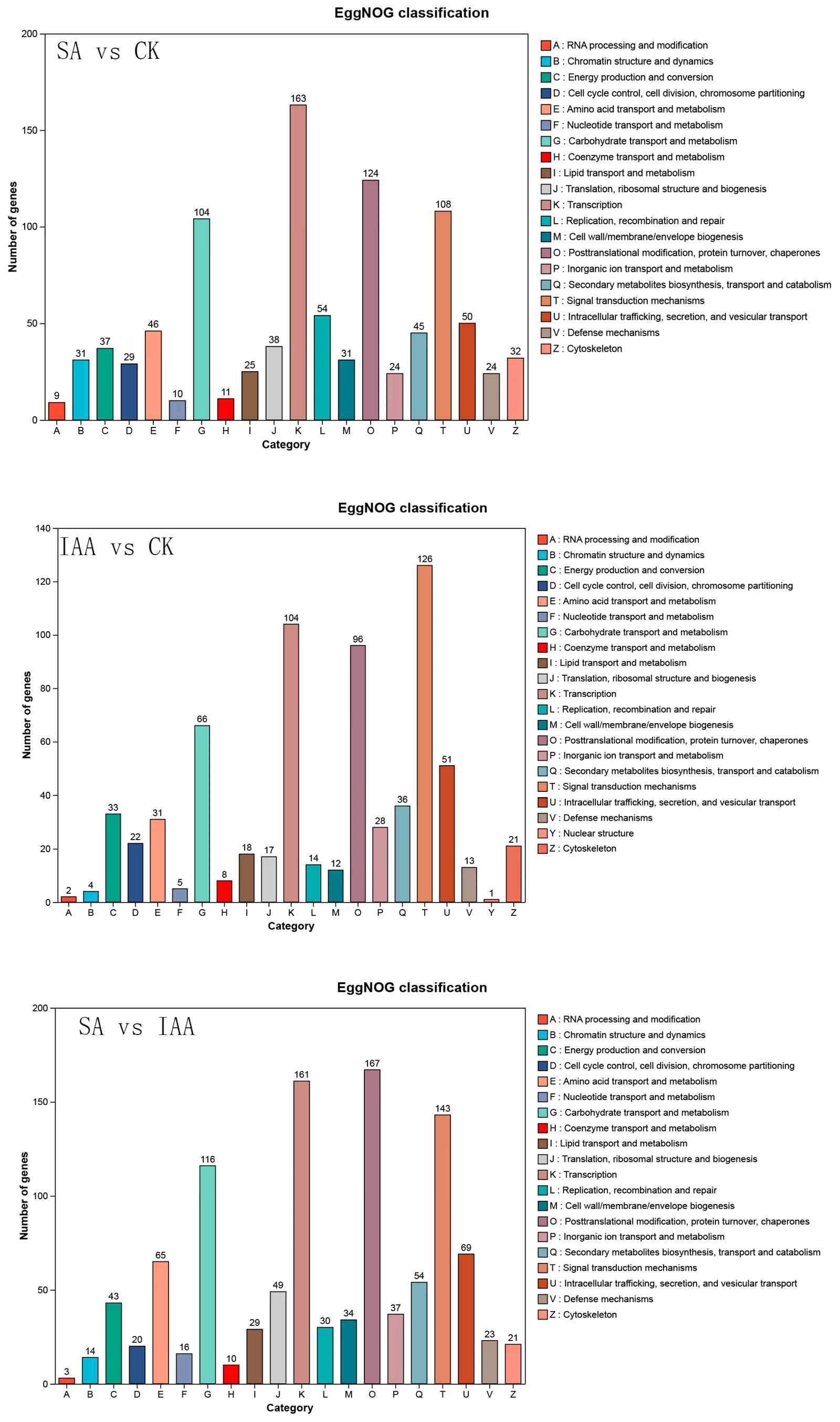
Functional cluster analysis based on the EggCOG database.

**Figure 8:**
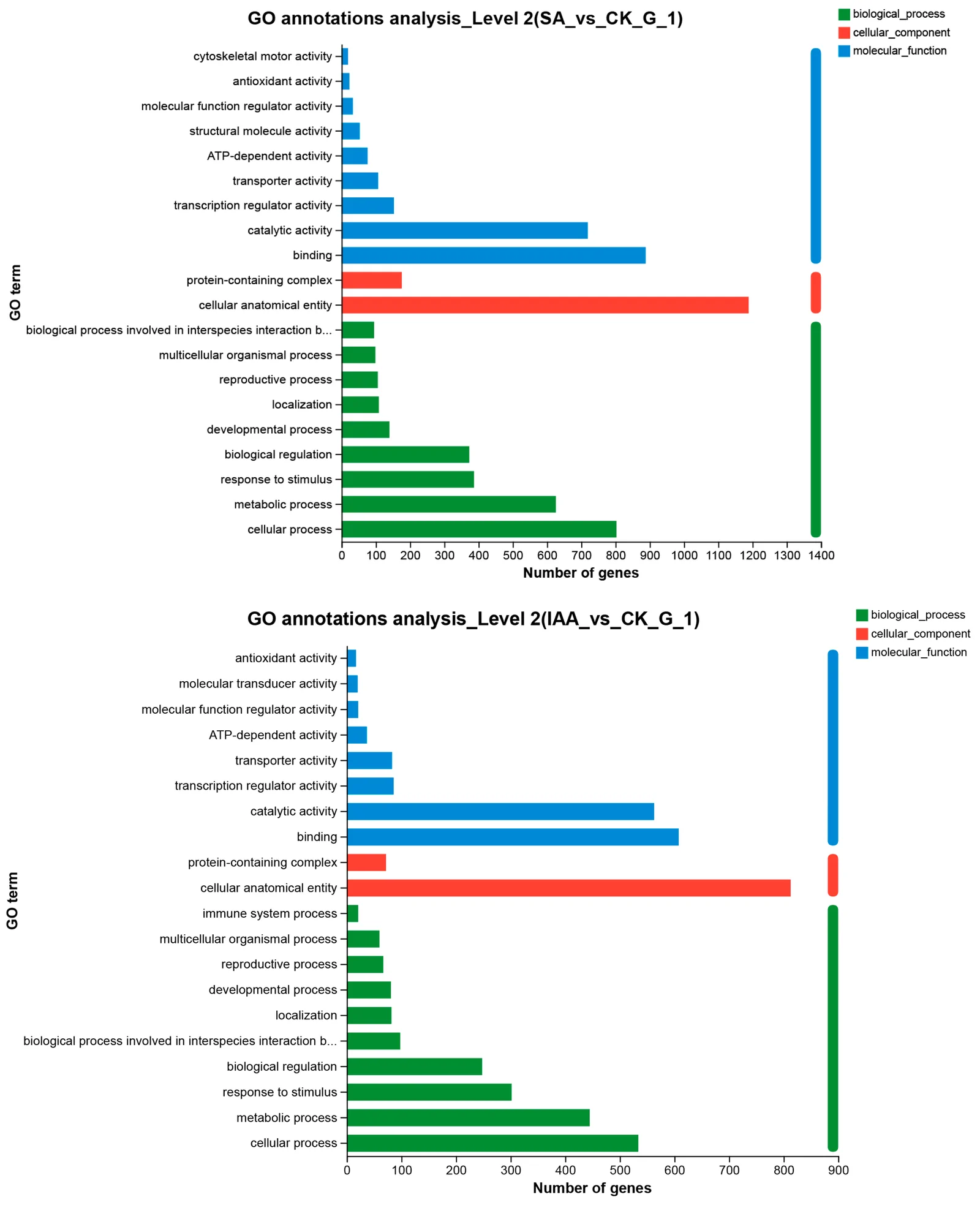

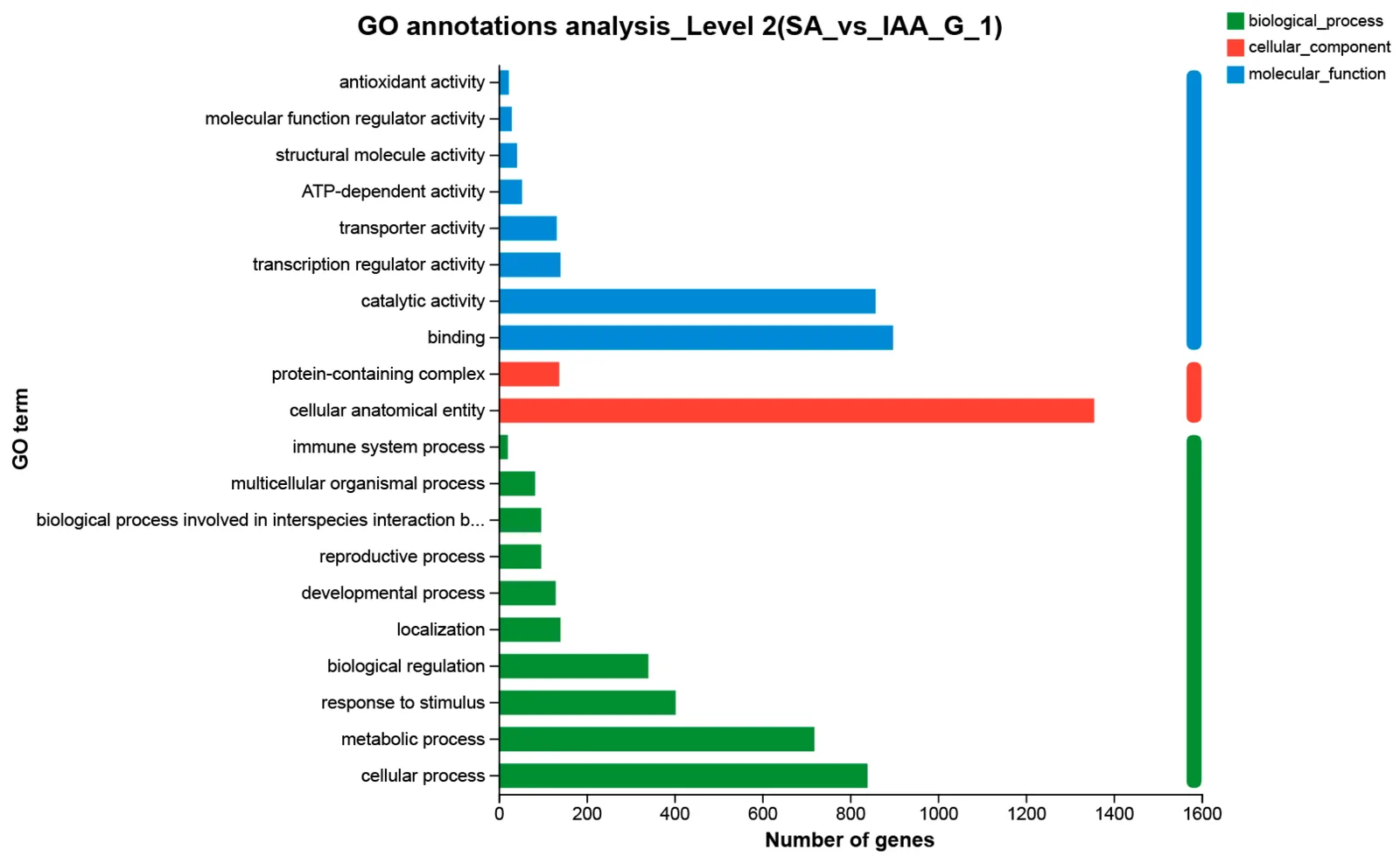
Functional annotation analysis based on the GO database.The ellipses (...) represent biological process involved in interspecies interaction between organisms.

**Figure 9:**
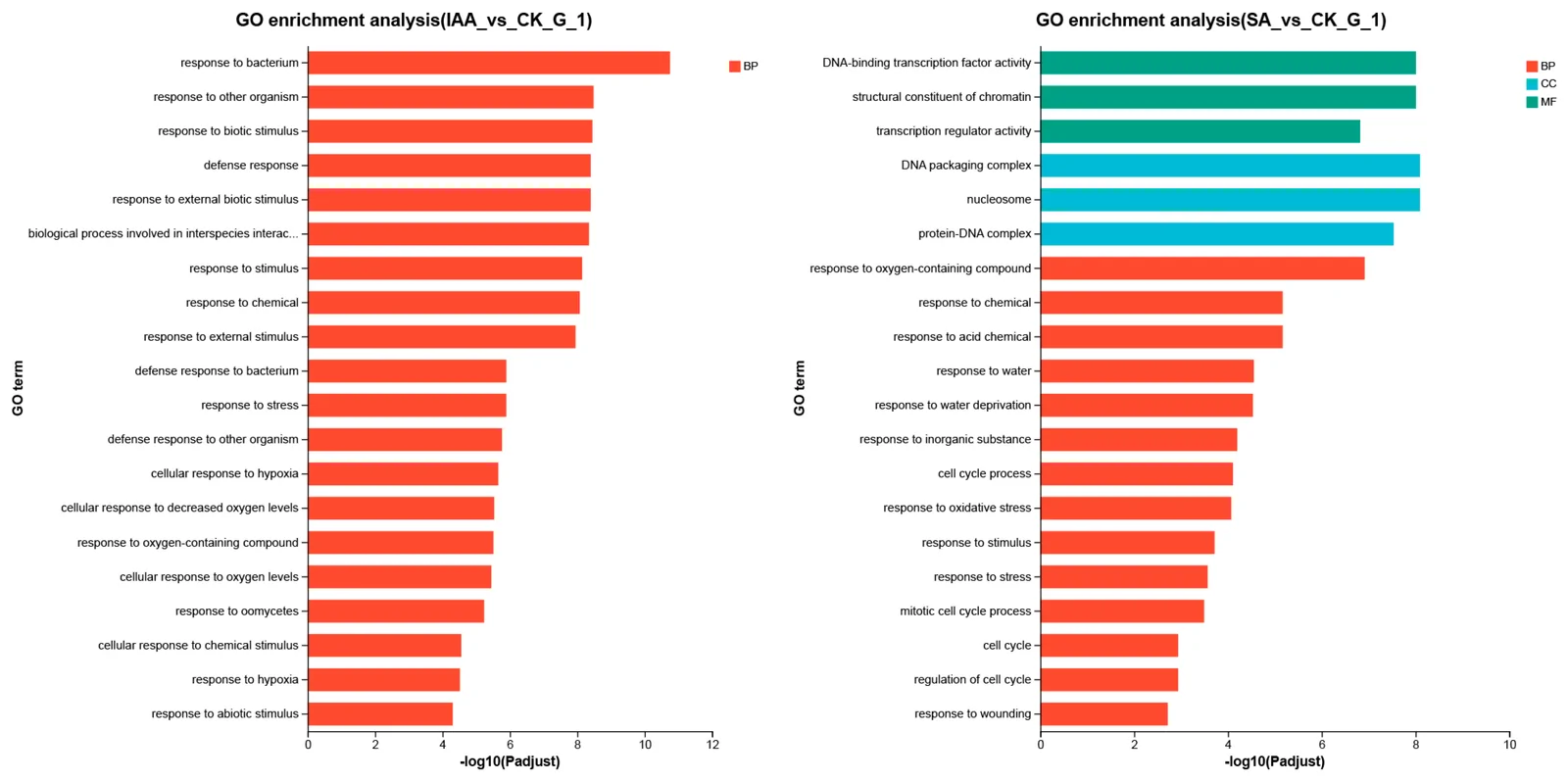
Significant GO functional enrichment analysis.The ellipses (...) represent biological process involved in interspecies interaction between organisms.

**Figure 10:**
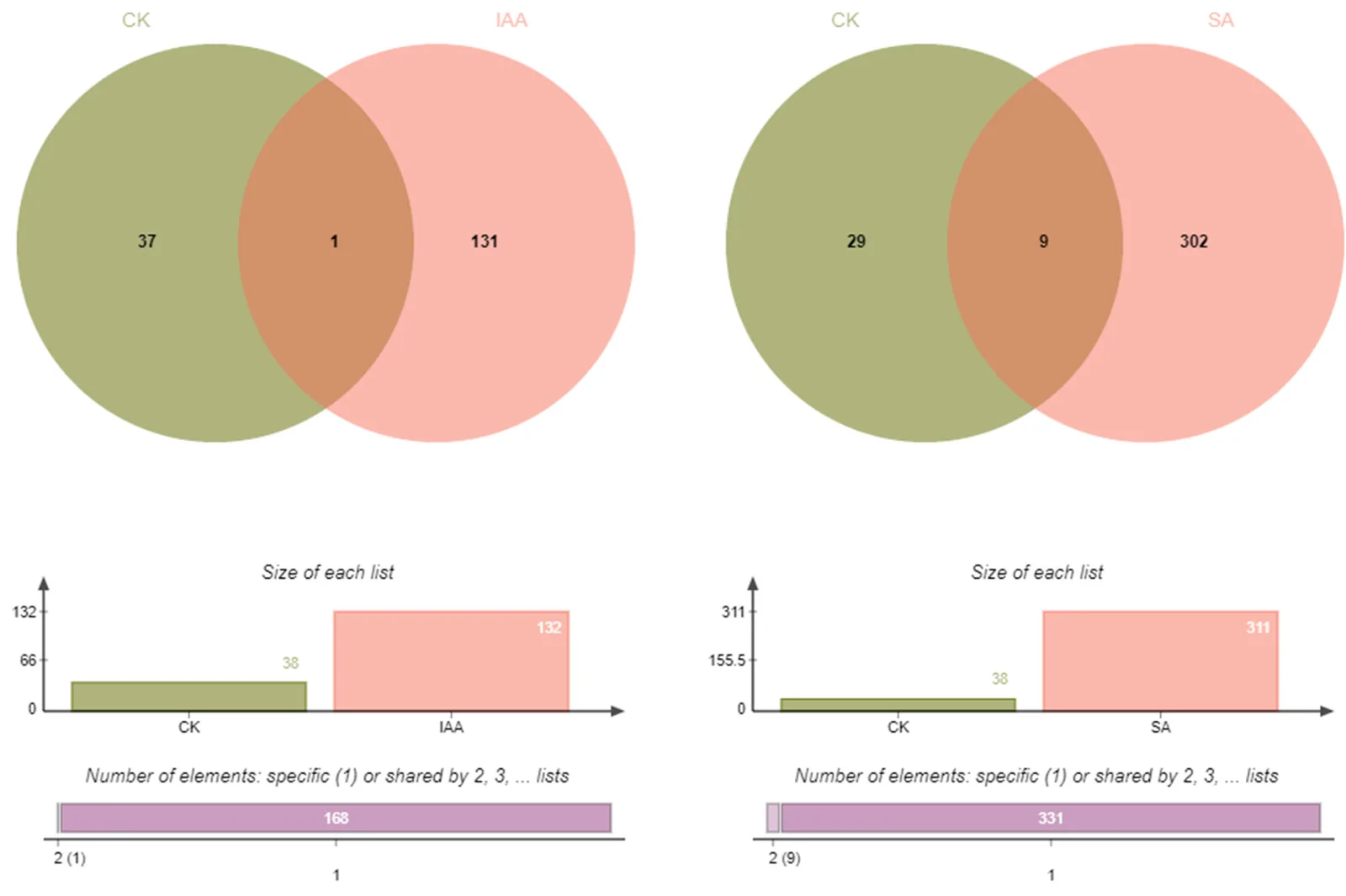
Screening results of DEGs under exogenous IAA and SA treatments.

**Figure 11:**
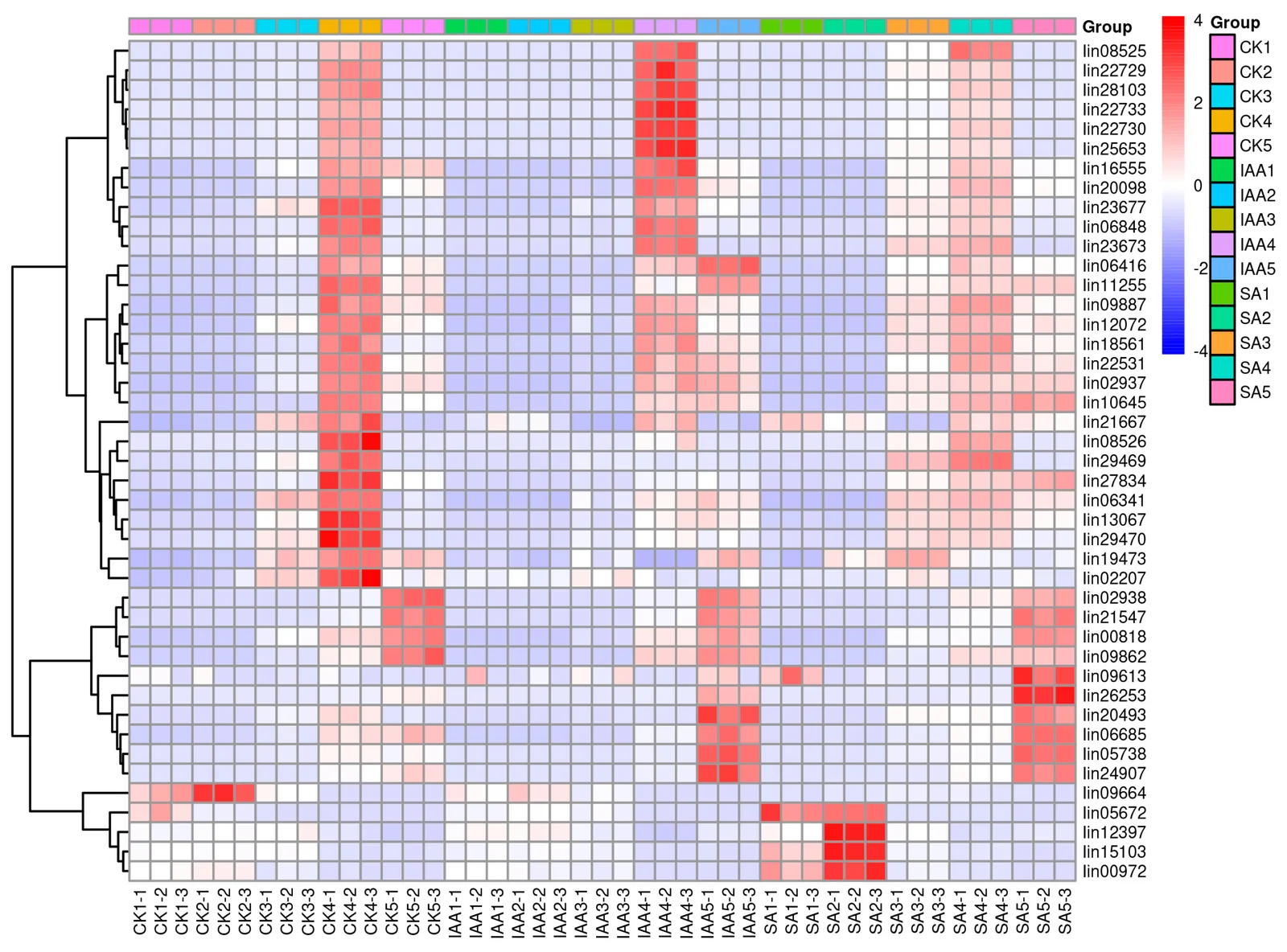
Heatmap of FPKM values of screened genes in response to exogenous IAA and SA.

**Figure 12:**
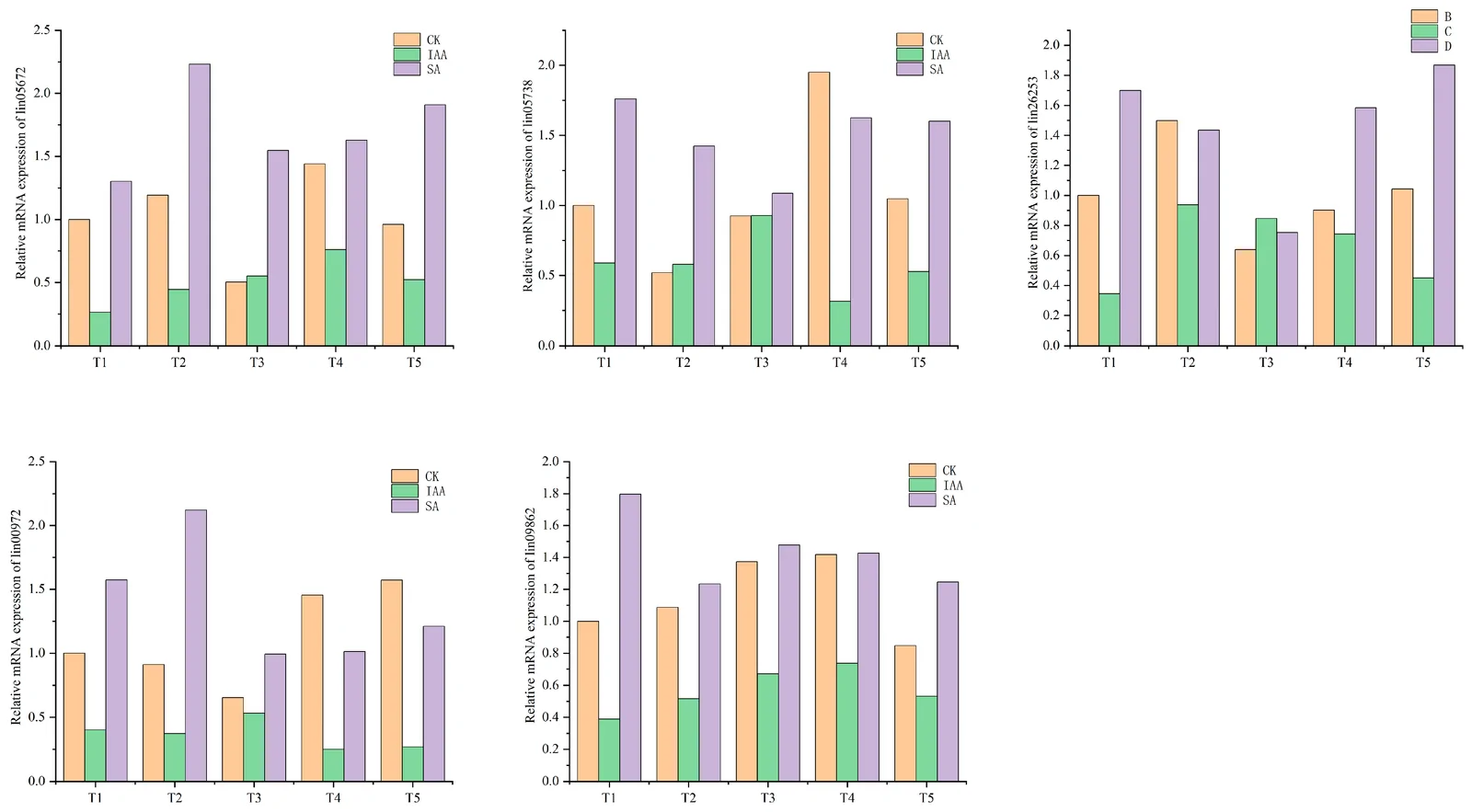
Relative mRNA expression levels of candidate genes in different treatments.

**Figure 13:**
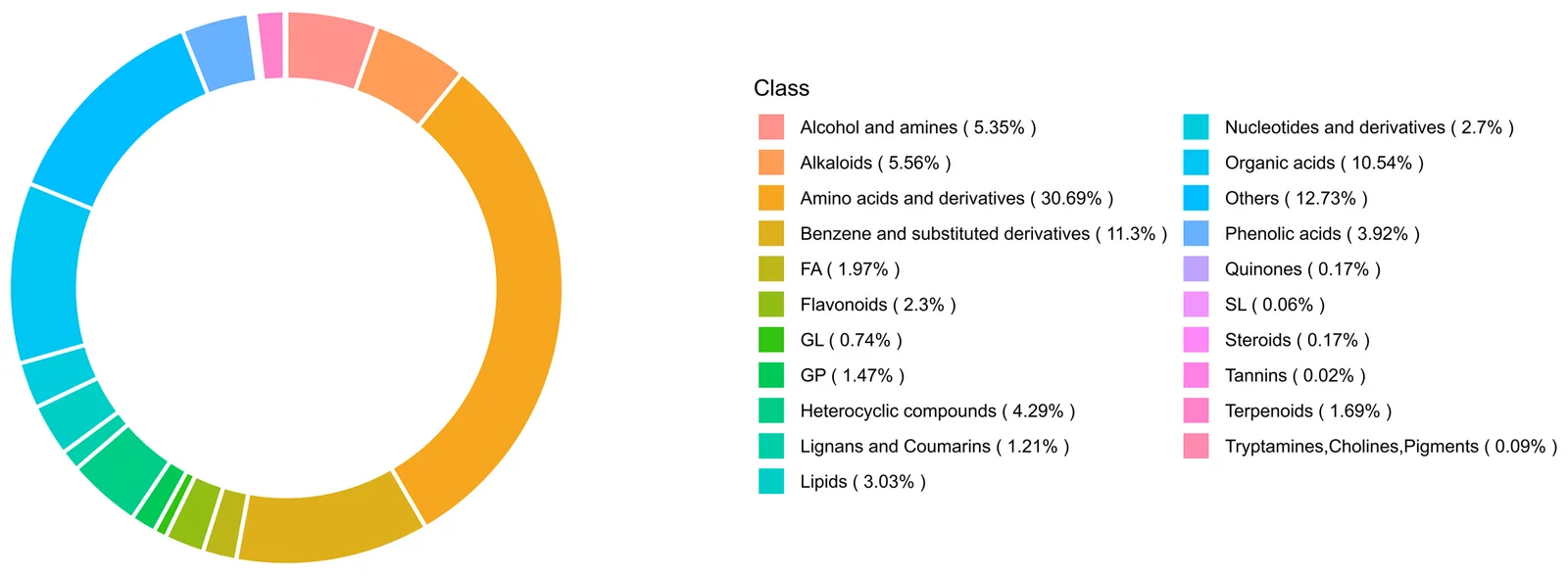

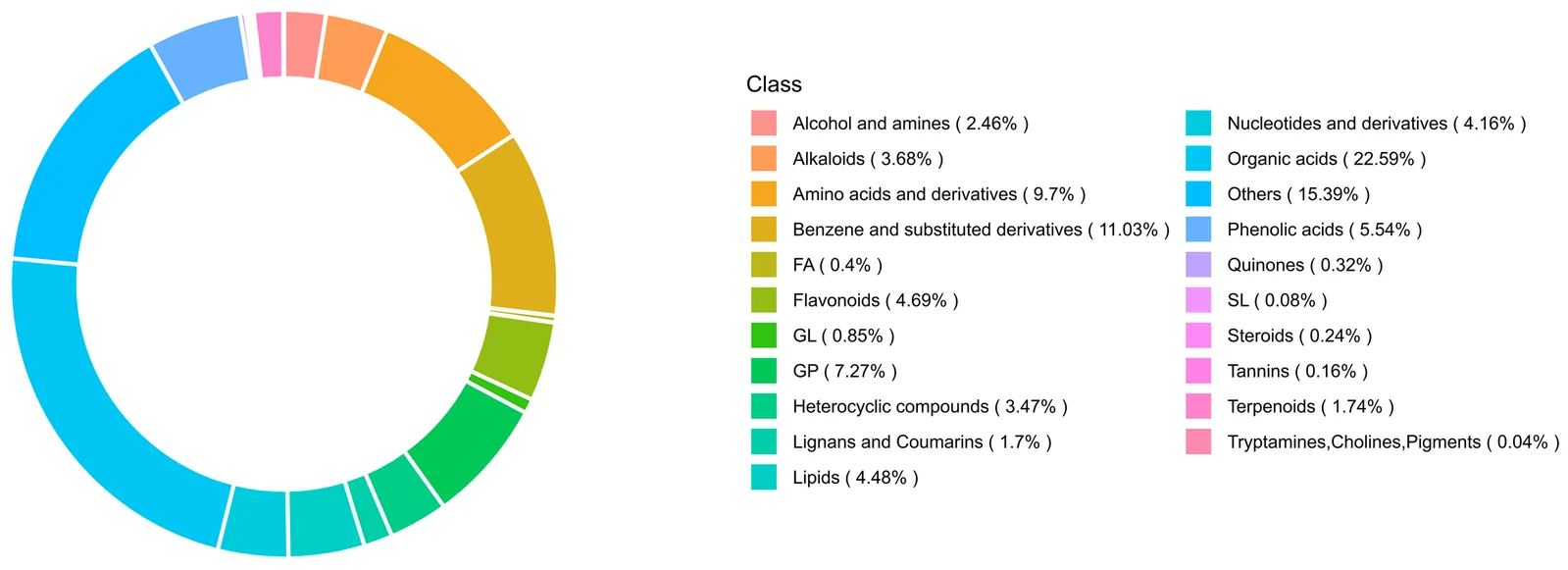
Classification of metabolites detected in positive ion (POS) and negative ion (NEG) modes.

**Figure 14:**
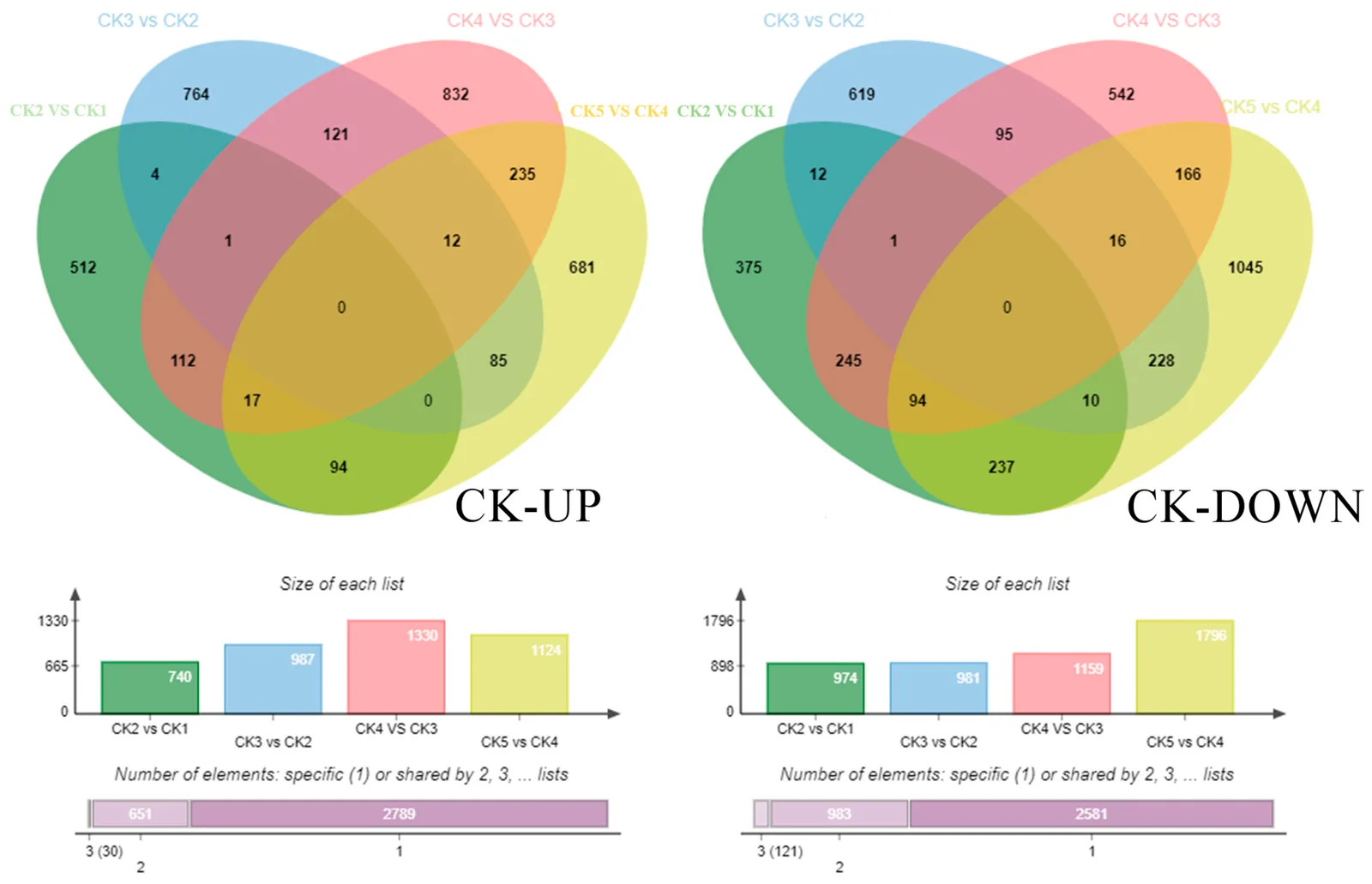
Screening of metabolites within the CK group.

**Figure 15:**
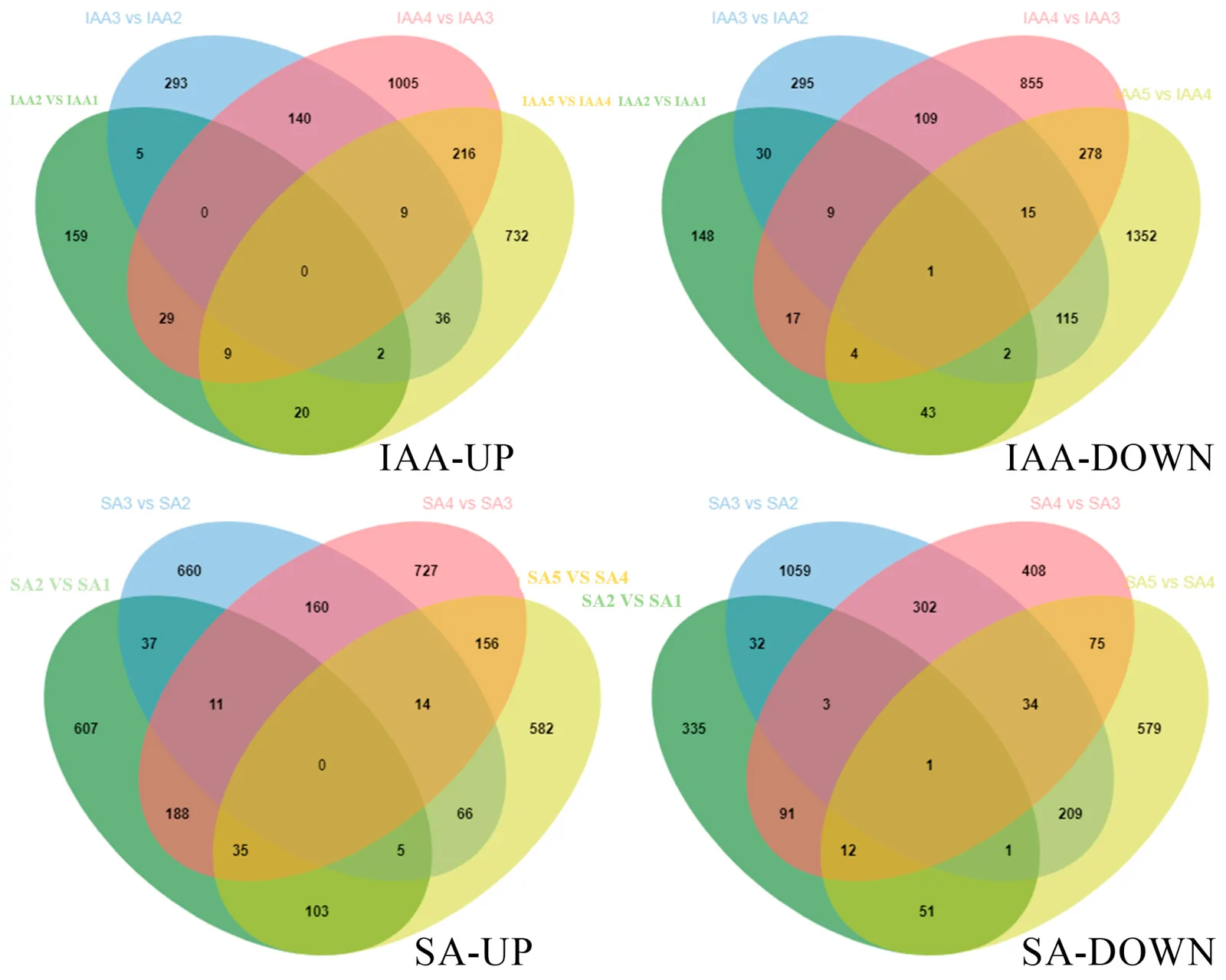
Screening of differentially accumulated metabolites (DAMs) within exogenous IAA- and SA-treated groups.

**Figure 16:**
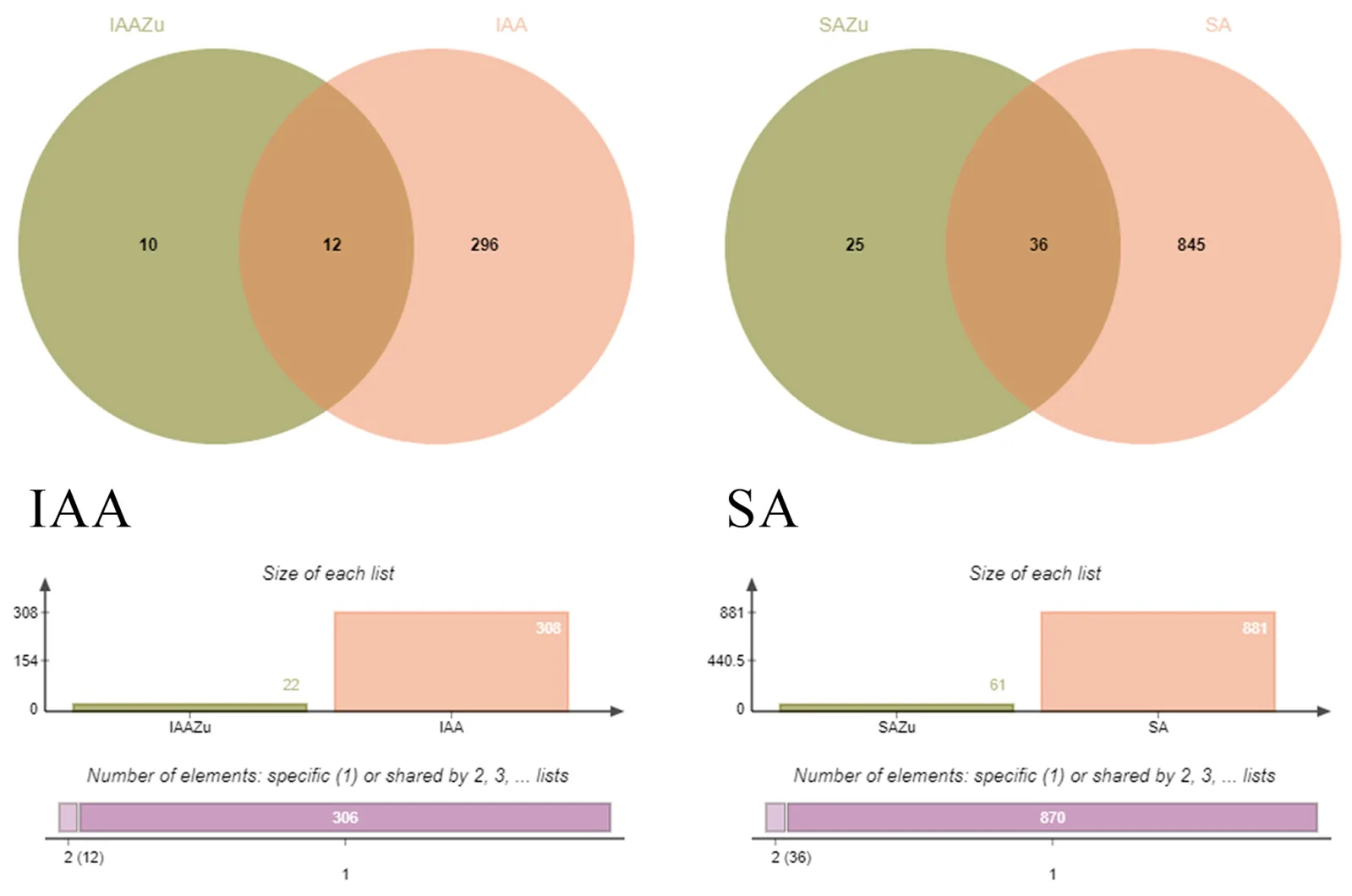
Compilation of differentially accumulated metabolites under exogenous IAA and SA treatments.

**Figure 17:**
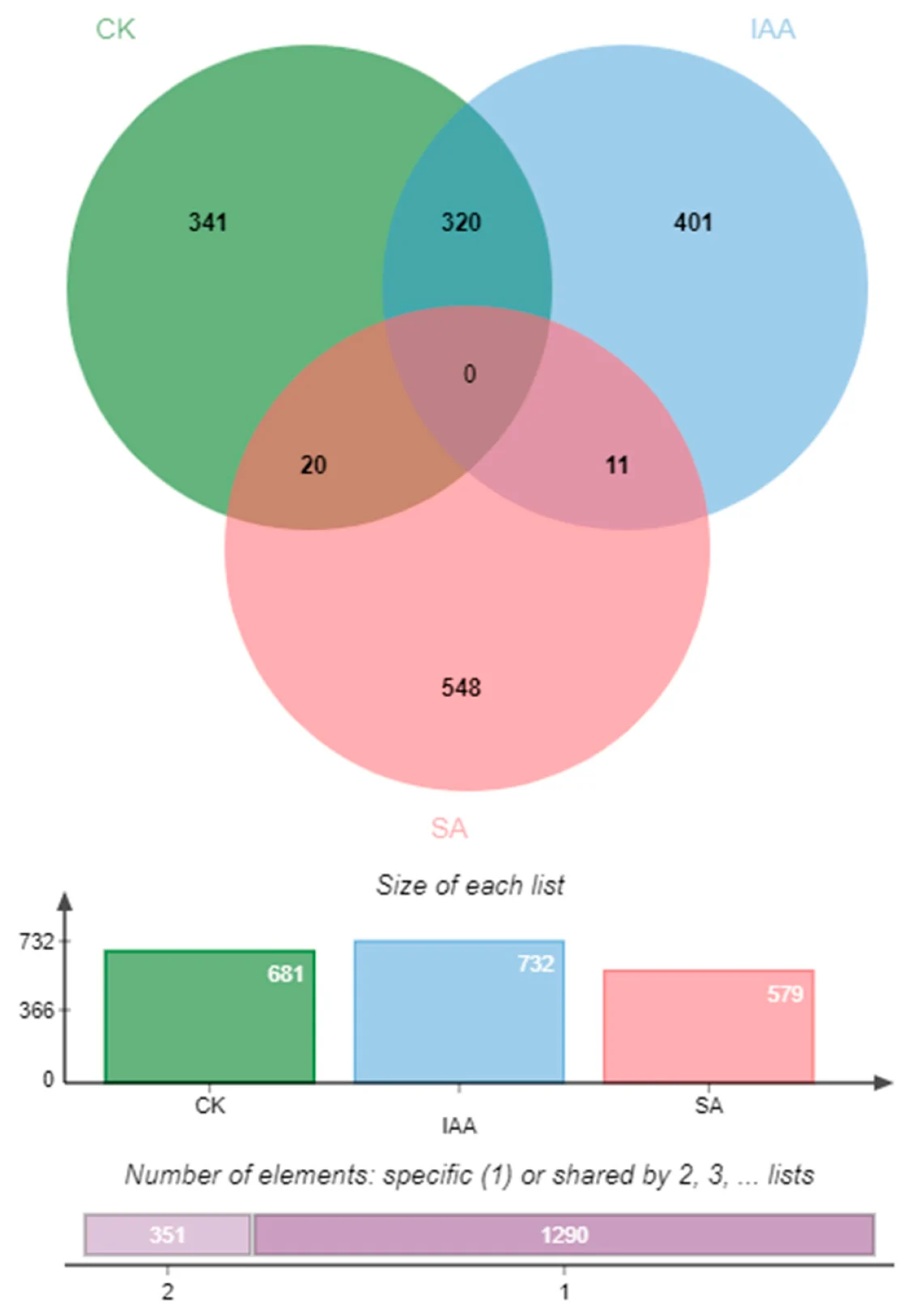
Screening of metabolites at stage T5.

**Figure 18:**
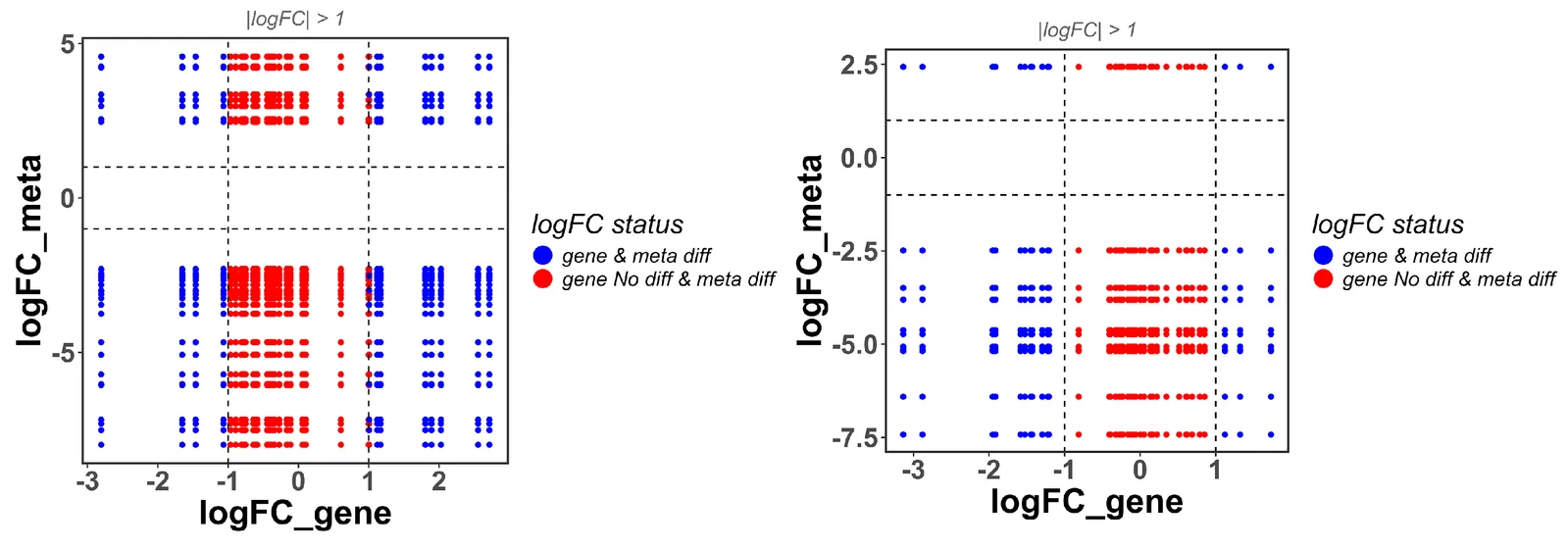
Nine-quadrant plot of screened genes and metabolites.

**Figure 19:**
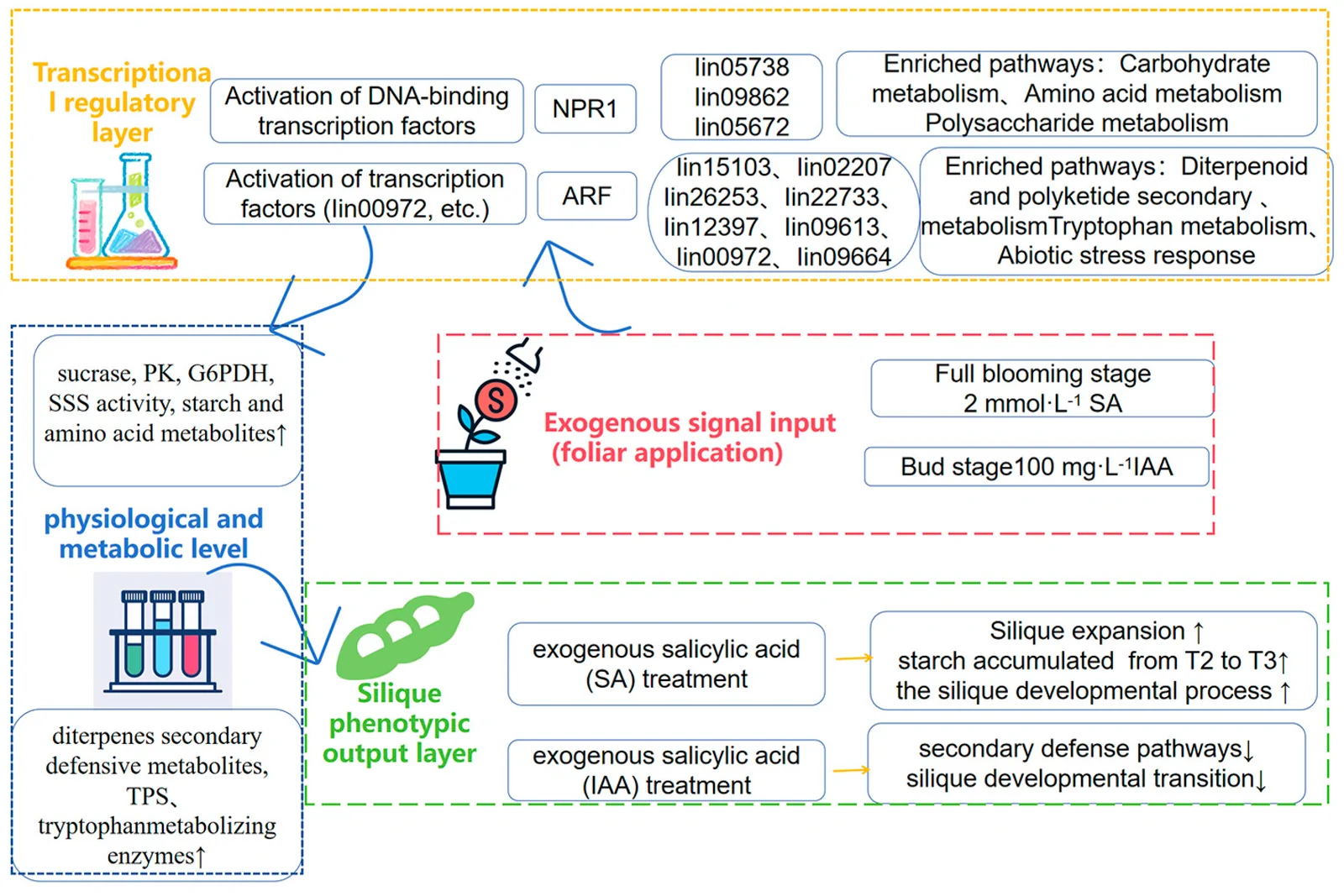
Proposed model for the regulatory effects of exogenous SA and IAA treatments on silique development in *I. indigotica*. This schematic integrates physiological, transcriptomic, and untargeted metabolomic data to illustrate putative response pathways underlying silique development upon exogenous SA and IAA application. Note that SA and IAA were sprayed at different developmental stages; the observed responses represent the combined effects of hormone treatment and application timing, rather than being solely attributable to hormone identity. Upward arrows indicate promotion, and downward arrows indicate inhibition.

**Table 1:** Sample collection times and numbers.

Period	Developmental Stage	Sampling Date	IAA Group ID	SA Group ID	CK Group ID
T1	Silique formation	23 May	IAA1	SA1	CK1
T2	Silique growth	31 May	IAA2	SA2	CK2
T3	Silique expansion	12 June	IAA3	SA3	CK3
T4	Silique browning maturation	2 July	IAA4	SA4	CK4
T5	Silique full maturation	22 July	IAA5	SA5	CK5

Note: T1–T5 represent five successive developmental phases of *Isatis indigotica* siliques.

**Table 2:** PCR primer sequences.

Primer ID	Nucleotide Sequence (5′-3′)		Tm Value	GC Content (%)	Product Length (bp)
*Iin05672*	sense	GATTGCTAGCACCACTTCTCTTG	58.8	47.8	150
	antisense	CGAAGAAGAACGGAATAGGTCTC	59.4	47.8	
*Iin26253*	sense	CGATCCATTCTCGCTTGACAT	60	47.6	218
	antisense	CGCTTATCTGAAGTATGTTGCC	57.3	45.5	
*Iin09862*	sense	CAACATCTGCTAGAAGAGCATACG	59.2	45.8	156
	antisense	TGTCCCAATACAACTTGTCTAGAGC	59.9	44	
*Iin05738*	sense	CCAGAAGCCGAACAGTCTCAG	59.8	57.1	213
	antisense	ATCTTGGCATTGAGCTAGCAAG	59.3	45.5	
*Iin00972*	sense	ATTACCAACAGCTTTCACATGC	57.3	40.9	162
	antisense	GCAATCTCTTCACAGTCGATACA	57.2	43.5	
*Actin*	sense	GACAATGGAACTGGAATGGTGA	59.6	45.5	145
	antisense	GAGCTTCATCACCAACGTAAGC	59.3	50	

## Data Availability

The raw experimental data supporting the findings of this study are openly available in Zenodo at https://doi.org/10.5281/zenodo.22822088.

## Abbreviations

The following abbreviations are used in this manuscript:

SA	Salicylic acid
IAA	Indole-3-acetic acid
PK	Pyruvate kinase
G6PDH	Glucose-6-phosphate dehydrogenase
SSS	Soluble starch synthase
DA-6	Diethyl aminoethyl hexanoate
BR	Brassinosteroid
GA	Gibberellin
MeJA	Methyl jasmonate
DPA	Days post-anthesis
DEGs	Differentially expressed genes
DEMs	Differentially expressed metabolites
SP	Soluble protein
CLL	Cellulose
SS	Soluble sugar
RS	Resistant starch
SS-I	Sucrose synthase (cleavage direction, SS-I)
SS-II	Sucrose synthase (synthetic direction, SS-II)
SBE	Starch-branching enzyme
ABA	Abscisic acid
